# Feline Diabetes Is Associated with Deficits in Markers of Insulin Signaling in Peripheral Tissues

**DOI:** 10.3390/ijms252313195

**Published:** 2024-12-08

**Authors:** Souvik Patra, Chantal J. McMillan, Elisabeth R. Snead, Amy L. Warren, Kevin Cosford, Prasanth K. Chelikani

**Affiliations:** 1 School of Veterinary Medicine, Texas Tech University, 7671 Evans Drive, Amarillo, TX 79106, USA; soupatra@ttu.edu; 2 Faculty of Veterinary Medicine, University of Calgary, 3280 Hospital Dr. NW, Calgary, AB T2N 4Z6, Canada; alwarren@ucalgary.ca; 3 Department of Small Animal Clinical Sciences, Western College of Veterinary Medicine, University of Saskatchewan, Saskatoon, SK S7N 5B4, Canada; ecs212@mail.usask.ca (E.R.S.); klc214@mail.usask.ca (K.C.)

**Keywords:** cats, insulin resistance, incretin signaling

## Abstract

Like humans, cats have a strong relationship between decreasing insulin sensitivity and the development of diabetes with obesity. However, the underlying molecular mechanisms of impaired insulin secretion and signaling in cats remain largely unknown. A total of 54 client-owned nondiabetic lean (*n* = 15), overweight (*n* = 15), and diabetic (*n* = 24) cats were included in the study. The pancreas, liver, and skeletal muscle were quantified for mRNA and protein abundances of insulin and incretin signaling markers. Diabetic cats showed increased liver and muscle adiposity. The pancreas of diabetic cats had decreased transcript abundances of insulin, insulin receptor, insulin-receptor substrate (IRS)-1, glucose transporters (GLUT), and protein abundance of mitogen-activated protein kinase. In treated diabetics, protein abundance of glucagon-like peptide-1 and glucose-dependent insulinotropic peptide receptors, total and phosphorylated Akt, and GLUT-1 were increased in the pancreas, whereas untreated diabetics had downregulation of markers of insulin and incretin signaling. In the muscle and liver, diabetic cats had reduced mRNA abundances of insulin receptor, IRS-1/2, and phosphatidylinositol-3-kinase, and reduced protein abundances of GLUT-4 and phosphatidylinositol-3-kinase-p85α in muscle. We demonstrate that feline diabetes is associated with ectopic lipid deposition in the liver and skeletal muscle, deficits in insulin synthesis and incretin signaling in the pancreas, and impaired insulin signaling in the muscle and liver. These findings have implications for understanding the pathophysiological mechanisms of obesity and diabetes in humans and pets.

## 1. Introduction

The pathophysiological mechanisms of obesity and diabetes have been traditionally studied using rodent models under controlled environments; however, less attention has been given to companion animal models of these conditions that might better simulate the human disease [1]. Diabetes mellitus (DM) is a common endocrinopathy in cats [2], with 80% of cats considered to have a disease comparable to type 2 diabetes mellitus (T2DM) in humans [3,4,5,6], characterized by insulin resistance (IR) and decreased insulin secretion. Common risk factors causing IR in cats and humans include obesity, treatment with pharmacological agents that cause decreased insulin sensitivity, endocrinopathies associated with insulin resistance (i.e., hypersomatotropism), increasing age, and physical inactivity [7,8,9]. Like humans, obesity is a significant risk factor for diabetes in cats, with up to 35% of cats classified as overweight or obese in the United States [10]. The effects of increasing body weight on insulin sensitivity are well documented in cats. For each 1–1.9 kg increase in body weight, there is a 30% [11] to 50% [12] reduction in insulin sensitivity. Obese cats appear to compensate for reduced peripheral insulin sensitivity by lowering hepatic glycogenolysis and endogenous glucose production [13]. Furthermore, sustained hyperglycemia that mimics diabetic glucose levels has been shown to cause a significant beta-cell loss in cats [14] and in human β-cells [15,16], and transcripts for insulin signaling in muscle tissue were reported to be decreased in obese cats [17,18] and humans [19]. Though feline diabetes shares many features with spontaneous type 2 diabetes in humans [1,20], the underlying molecular mechanisms that predispose cats to decreased insulin secretion and insulin resistance remain unclear.

In humans, insulin secretion and signaling are well-characterized processes critical for glucose homeostasis. Insulin secretion from pancreatic beta cells is triggered by glucose uptake via transporters (GLUT1, GLUT2), with GLUT2 being particularly essential for glucose-stimulated insulin secretion. Insulin secretion is further amplified by glucose, metabolic substrates, and neuroendocrine signals [21,22]. Of the endocrine signals, the incretin hormones glucagon-like peptide-1 (GLP-1) and glucose-dependent insulinotropic peptide (GIP) enhance insulin secretion, promote beta-cell survival, and modulate glucagon release [23,24]. The molecular events in the regulation of insulin secretion from the pancreas are unknown in cats. The binding of insulin to its cognate receptor results in autophosphorylation and subsequent recruitment of insulin receptor substrate (IRS) to the activated receptor and activation of phosphoinositide-dependent kinase-1, which in turn activates p85/p110 phosphatidylinositol 3-kinase (PI3K) and Akt/PKB (protein kinase B), which subsequently trigger other downstream regulators to facilitate glucose uptake and storage, and lipid metabolism, in peripheral tissues [25,26]. IRS-1, IRS-2, and PI3K are mainly expressed in insulin-sensitive tissues such as the liver, skeletal muscle, and abdominal fat [17]. In humans and mice models, subjects with insulin resistance and obesity have decreased levels of IRS-1, IRS-2, and PI3K expression in several insulin-sensitive tissues [3,18,19], and of p42/p44 mitogen-activated protein (MAP) kinase in skeletal muscle [20,21]. Obese rats have also been shown to have ectopic fat deposition in the liver together with upregulation of acetyl-CoA carboxylase (ACC)—a key rate-limiting enzyme of fatty acid synthesis [22,23]. However, in cats, the mechanisms of insulin resistance are less understood. Obese cats were found to have decreased transcript levels of insulin signaling intermediates such as IRS-1/2 and PI3K in the liver and skeletal muscle [18] and decreased protein levels of GLUT-4 in skeletal muscle and adipose tissue [27], but tissue-specific signaling defects of other insulin signaling markers remain under explored. Thus, the molecular mechanism(s) of insulin secretion from the pancreas, and resistance to insulin action in peripheral tissues, remains poorly understood in cats.

We [28] and others [29,30] have shown that the feline gastrointestinal tract expresses transcripts [28] and immunopositivity [29,30] for the incretin hormones glucagon-like peptide-1 (GLP-1) and glucose-dependent insulinotropic peptide (GIP). The incretin effect was reported to be greatly decreased in human type 2 DM patients [31] and obese youth [32], in association with reductions in the insulinotropic potency of GLP-1 and in GIP-dependent insulin secretion [33]. The incretin effect in healthy cats does not appear to be as robust as that in humans and rodents, with insulin secretion being minimal after an oral glucose load, and is associated with increased GLP-1 but not GIP concentrations [34,35]. In previous reports, circulating GLP-1 concentrations were either decreased [36] or increased [37] in overweight and obese cats, and diabetic cats had greater plasma GLP-1 and GIP concentrations than lean cats [37]. However, the molecular mechanisms by which incretins regulate insulin secretion from feline pancreas are unknown. We hypothesized that incretin signaling in the pancreas, and insulin signaling in the liver and skeletal muscle, could be dysfunctional in overweight and diabetic cats. Our objectives were to determine the relative transcript and protein abundances of key intermediaries of insulin synthesis and incretin signaling in the pancreas, as well as insulin and incretin signaling in the skeletal muscle and liver, in a client-owned population of lean, overweight, and diabetic cats.

## 2. Results

### 2.1. Body Weight, Tissue Adiposity, and Blood Glucose and Fructosamine Were Increased in Diabetic Cats

Age and sex distribution did not differ among groups (Table 1). Compared with lean cats, overweight cats had significantly higher body weight (BW), body fat percentage, body mass index (BMI), and body condition score (BCS), whereas both untreated and treated diabetic groups had significantly increased BW, BCS, and blood glucose and fructosamine concentrations (Table 1). Compared with lean cats, liver fat percentage increased in the untreated diabetic group, and muscle fat percentage increased in the overweight and treated diabetic groups (Table 1). 

### 2.2. Key Markers of Insulin Synthesis and Signaling, Glucose Regulation, and Incretin Signaling Were Decreased in the Pancreas of Diabetic Cats

**Figure 1:**
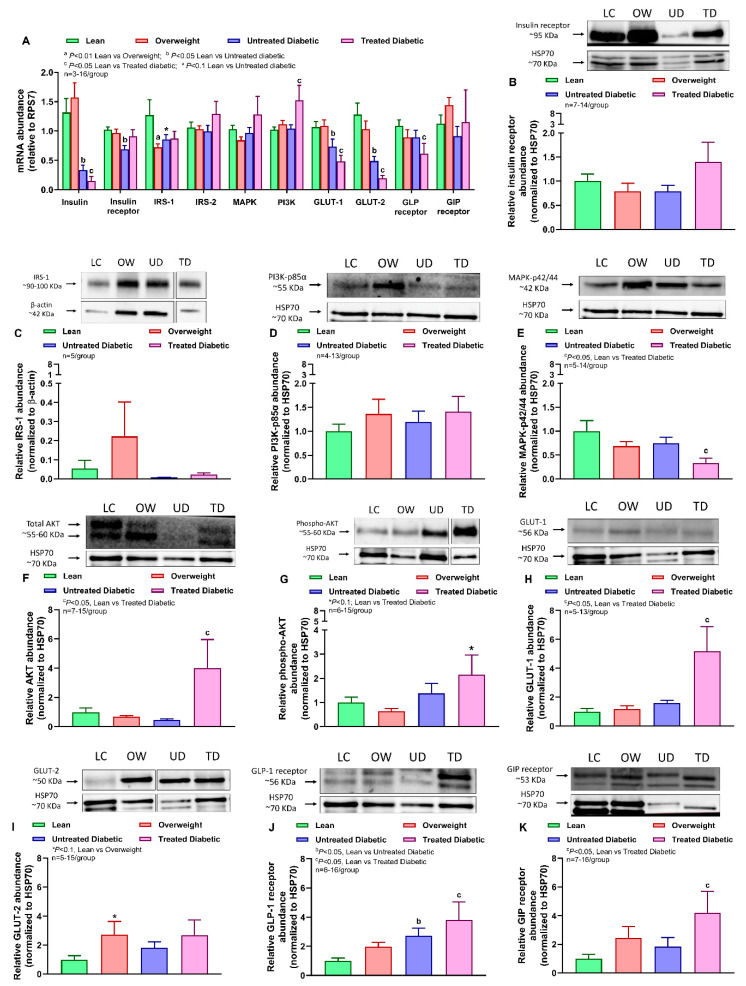
Differences among lean, overweight, and untreated and treated diabetic cats in pancreatic markers of insulin and incretin signaling. (**A**) Relative pancreatic mRNA abundances of insulin, insulin receptor, insulin receptor substrate (IRS)-1, insulin receptor substrate (IRS)-2, mitogen-activated protein kinase (MAPK)-3, phosphoinositide 3-kinase (PI3K), glucose transporter (GLUT)-1, glucose transporter (GLUT)-2, glucagon-like peptide (GLP)-1 receptor, and glucose insulinotropic peptide (GIP) receptor. Relative protein abundance in the pancreas of (**B**) insulin receptor, (**C**) insulin receptor substrate (IRS)-1, (**D**) phosphoinositide 3-kinase (PI3K), (**E**) mitogen-activated protein kinase (MAPK)-p42/44, (**F**) protein kinase B (AKT), (**G**) phosphorylated AKT, (**H**) glucose transporter (GLUT)-1, (**I**) glucose transporter (GLUT)-2, (**J**) glucagon-like peptide (GLP)-1 receptor, and (**K**) glucose insulinotropic peptide (GIP) receptor. Data were analyzed by one-way ANOVA with Benjamini–Hochberg corrections for multiple comparisons and a false discovery rate of 0.05. Values are mean ± SEM, *p* < 0.05.

In the pancreas, compared with lean cats, insulin mRNA abundance decreased in both the untreated and treated diabetic groups, whereas insulin receptor mRNA abundance decreased only in untreated diabetic cats (Figure 1A). IRS-1 but not IRS-2 mRNA abundance significantly decreased in the pancreas of overweight cats and tended to decrease in untreated diabetic cats compared with lean cats. Both GLUT-1 and GLUT-2 mRNA levels decreased in the pancreas of both diabetic groups. In treated diabetic cats, PI3K mRNA abundance was higher than in lean cats, and MAPK expression was unaltered among the groups. GLP-1 receptor mRNA abundance was lower in treated diabetic cats than in lean cats, whereas the GIP receptor mRNA levels remained unchanged (Figure 1A). Relative protein abundances of insulin receptor (Figure 1B, Supplementary Figures S1–S4), IRS-1 (Figure 1C, Supplementary Figure S5), and PI3K-p85α (Figure 1D, Supplementary Figure S6) in the pancreas did not differ among groups. In contrast to the mRNA levels, the protein abundance of MAPK-p42/44 decreased in the treated diabetic group (Figure 1E, Supplementary Figure S7), whereas total AKT and GLUT-1 protein abundance significantly increased, and phosphorylated AKT tended to increase, in treated diabetic cats compared with lean cats (Figure 1F–H, Supplementary Figures S8–S10 and S30). The ratio of phospho-AKT to total AKT tended to increase in the treated diabetic group compared with the lean group (lean: 1.44 ± 8.06; overweight: 1.2 ± 1.16; untreated diabetic: 1.47 ± 1.95; treated diabetic: 5.37 ± 1.76). GLUT-2 protein abundance tended to increase in overweight compared with lean cats (Figure 1I, Supplementary Figures S11 and S12). Contrary to the mRNA levels of incretins, the protein abundance of GLP-1 receptor increased in both untreated and treated diabetic cats (Figure 1J, Supplementary Figure S13), whereas GIP receptor protein levels increased only in the treated group (Figure 1K, Supplementary Figure S14), compared with lean cats. The sample size for each molecular target and treatment group is provided in Supplementary Table S3.

### 2.3. Markers of Insulin Signaling Were Decreased in the Muscle of Diabetic Cats

**Figure 2:**
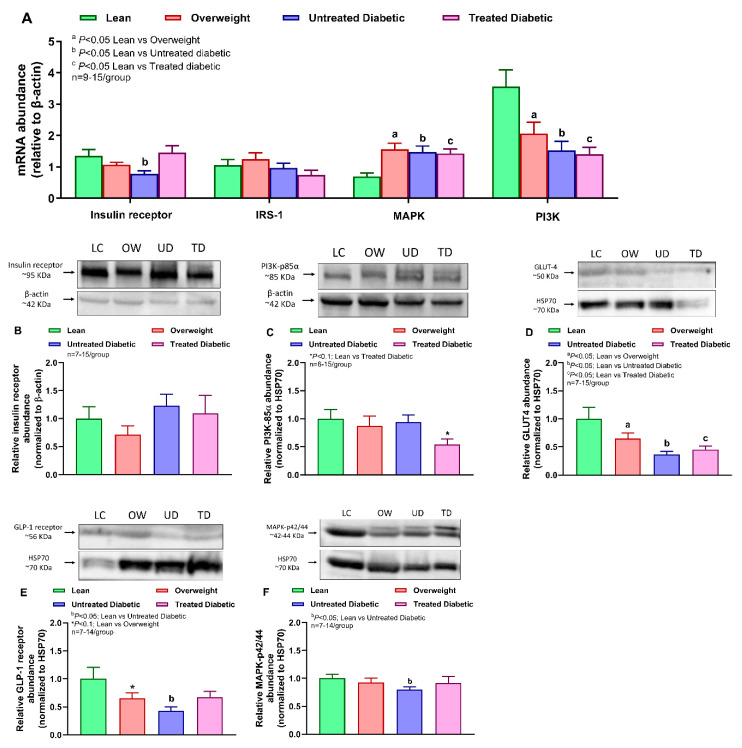
Differences among lean, overweight, and untreated and treated diabetic cats in muscle insulin signaling markers. (**A**) Relative muscle mRNA abundances of insulin receptor, insulin receptor substrate (IRS)-1, mitogen-activated protein kinase (MAPK)-3, and phosphoinositide 3-kinase (PI3K). Relative muscle protein abundances of (**B**) insulin receptor, (**C**) phosphoinositide 3-kinase (PI3K)-85α, (**D**) glucose transporter (GLUT)-4, (**E**) glucagon-like peptide (GLP)-1 receptor, and (**F**) mitogen-activated protein kinase (MAPK)-p42/44. Data were analyzed by one-way ANOVA with Benjamini–Hochberg corrections for multiple comparisons and a false discovery rate of 0.05. Values are mean ± SEM, *p* < 0.05.

Insulin receptor mRNA abundance decreased in muscle of untreated diabetic compared with lean cats (Figure 2A). The subsequent downstream regulators, such as PI3K mRNA abundance, decreased, whereas MAPK3 mRNA abundance increased in overweight and diabetic compared with lean cats, and the IRS-1 mRNA level remained unchanged (Figure 2A). However, protein abundance of insulin receptor did not differ among groups (Figure 2B, Supplementary Figure S15). Protein abundance of PI3K-p85α tended to decrease in the treated diabetic group compared with the lean group (Figure 2C, Supplementary Figure S16). GLUT-4 protein abundance decreased in the overweight and diabetic groups compared with the lean group (Figure 2D, Supplementary Figure S17), and GLP-1 receptor abundance decreased in untreated diabetic cats but showed a tendency to decrease in overweight cats compared with lean cats (Figure 2E, Supplementary Figure S18). The protein abundance of MAPK-p42/44 decreased only in untreated diabetic cats compared with lean cats (Figure 2F, Supplementary Figure S31).

### 2.4. Markers of Glucose Transporters and Fatty Acid Synthesis Decreased in the Liver of Diabetic Cats

**Figure 3:**
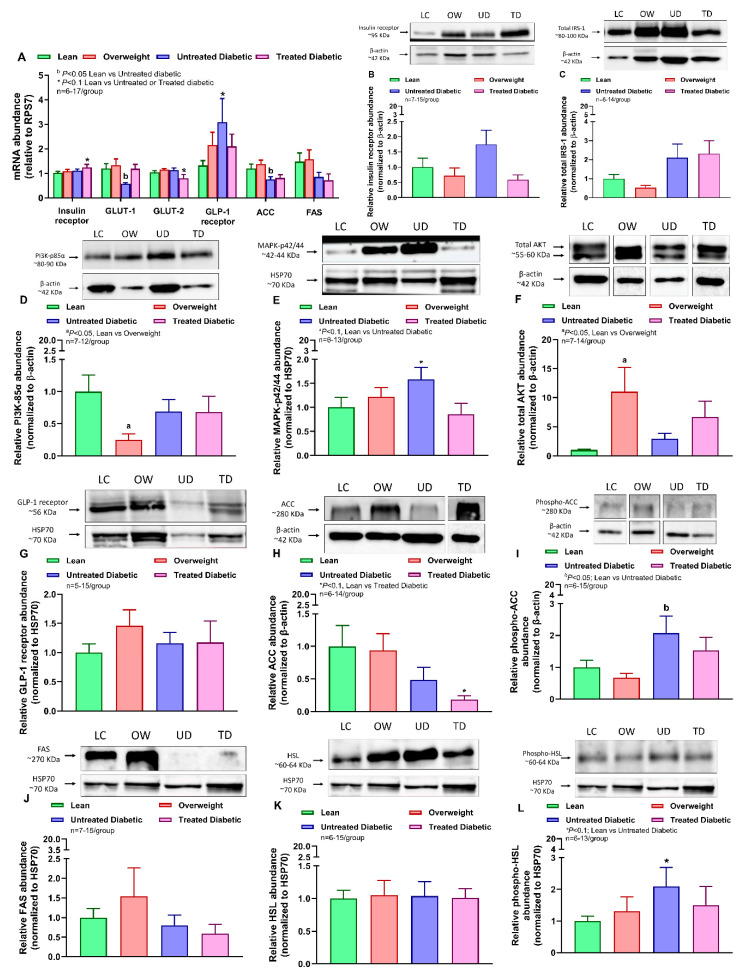
Differences among lean, overweight, and untreated and treated diabetic cats in liver insulin signaling markers. (**A**) Relative liver mRNA abundance of insulin receptor, glucose transporter (GLUT)-1, glucose transporter (GLUT)-2, glucagon-like peptide (GLP)-1 receptor, acetyl-CoA carboxylase (ACC), and fatty acid synthase (FAS). Relative protein abundances of (**B**) insulin receptor, (**C**) insulin receptor substrate (IRS)-1, (**D**) phosphoinositide 3-kinase (PI3K), (**E**) mitogen-activated protein kinase (MAPK)-p42/44, (**F**) protein kinase B (AKT), (**G**) glucagon-like peptide (GLP)-1 receptor, (**H**) acetyl-CoA carboxylase, (**I**) phosphorylated ACC, (**J**) fatty acid synthase (FAS), (**K**) hormone sensitive lipase (HSL), and (**L**) phosphorylated HSL. Data were analyzed by one-way ANOVA with Benjamini–Hochberg corrections for multiple comparisons and a false discovery rate of 0.05. Values are mean ± SEM, *p* < 0.05.

In the liver, insulin receptor abundance tended to increase in treated diabetic compared with lean cats; GLUT-1 mRNA decreased in untreated diabetic cats, and GLUT-2 tended to decrease in the treated diabetic group. Furthermore, the mRNA abundance of GLP-1 receptor tended to increase, whereas ACC decreased, in the untreated diabetic group compared with the lean group (Figure 3A). The relative protein abundances of insulin receptor (Figure 3B, Supplementary Figure S19) and IRS-1 (Figure 3C, Supplementary Figure S20) did not differ among groups. Notably, relative protein abundances of PI3K-p85α decreased (Figure 3D, Supplementary Figure S21), whereas total AKT increased, in overweight cats (Figure 3F, Supplementary Figures S23 and S24) compared with lean cats. In untreated diabetic cats, the relative protein abundance of MAPK-p42/44 tended to increase (Figure 3E, Supplementary Figure S22) compared with lean cats. In treated diabetic cats, total ACC tended to decrease (Figure 3H, Supplementary Figure S26), but phosphorylated ACC protein abundance significantly increased in untreated diabetic cats (Figure 3I, Supplementary Figure S32) compared with lean cats, and the ratio of phospho-ACC to total ACC did not differ among groups (lean: 11.79 ± 29.26; overweight: 2.31 ± 8.39; untreated diabetic: 7.24 ± 12.4; treated diabetic: 14.06 ± 7.94). The relative protein abundances of GLP-1 receptor (Figure 3G, Supplementary Figure S25), FAS (Figure 3J, Supplementary Figure S27), and HSL (Figure 3K, Supplementary Figures S28 and S29) did not differ from lean cats. However, phosphorylated HSL protein abundance tended to increase in untreated diabetic cats (Figure 3L, Supplementary Figure S33), and similarly, the ratio of phospho-HSL to total HSL tended to increase in untreated diabetic cats compared with lean cats (lean: 1.16 ± 0.79; overweight: 1.99 ± 1.05; untreated diabetic: 2.96 ± 1.03; treated diabetic: 2.08 ± 1.1).

## 3. Discussion

Feline diabetes is postulated to be associated with insulin resistance in peripheral tissues, with a clear correlation with increasing fat mass in cats, in which impacts are greatest in the males. However, the underlying molecular mechanisms of insulin resistance and incretin signaling remain largely unknown. Our study provides several important insights into the potential mechanisms of insulin resistance in overweight and diabetic cats. First, we demonstrate that diabetic cats exhibited a higher relative adiposity in their livers and muscle compared with lean cats, while overweight cats displayed increased adiposity in their muscle. Second, in the pancreas of both diabetic groups, the transcript abundance of regulatory markers of insulin secretion including insulin, GLUT1, and GLUT2 decreased. In contrast, in treated diabetics, the protein abundances of total Akt, phosphorylated Akt, GLP-1 and GIP receptors, and GLUT1 increased, but MAPK-p42/44 decreased in the pancreas. Third, in untreated diabetics, the skeletal muscle exhibited indices of impaired insulin and incretin signaling, which was supported by the downregulation of transcripts of insulin receptor and PI3K transcripts and decreased protein abundances of GLP-1 receptor and GLUT-4, and potentially increased hepatic lipid flux, with upregulation of both rate-limiting lipogenic (phosphorylated ACC) and lipolytic (phosphorylated HSL) enzymes. Fourth, diabetic cats had reduced mRNA abundance of genes linked to insulin signaling in the muscle and liver, including the insulin receptor, IRS-1/2, and PI3K, and reduced protein abundances of GLUT-4 and PI3K-p85α in the muscle. Together, these findings indicate that feline diabetes is associated with deficits in insulin synthesis and incretin signaling in the pancreas and impaired insulin signaling in the muscle and liver.

In humans, obesity is known to be associated with nonalcoholic fatty liver disease, where the accumulation of triglycerides in the liver correlates with insulin resistance in various tissues, including the liver, muscle, and adipose tissue [38]. We observed that, similarly to the human reports, untreated diabetic cats had increased percentages of liver fat, and overweight and treated diabetic cats had increased percentages of muscle fat. In the liver, we noted that untreated diabetics had decreased transcript abundance and treated diabetics had decreased protein abundance of the rate-limiting lipogenic enzyme acetyl-CoA carboxylase, suggestive of compensatory downregulation, and no changes in transcripts and/or proteins of fatty acyl synthase. The increases in the protein abundances of both the phosphorylated ACC (lipogenic) and HSL (lipolytic) in the livers of untreated diabetic cats suggest that, despite a reduction in ACC at the mRNA level, both lipogenesis and lipolysis are likely upregulated, contributing to a greater lipid flux and metabolic dysregulation in the liver. The apparent paradox indicates that the key regulatory control of lipogenesis in the feline liver and muscle may be further downstream of acetyl-CoA carboxylase and fatty acyl synthase. Nonetheless, these findings strongly indicate that ectopic lipid deposition in the liver and muscle is likely associated with impaired glucose homeostasis, particularly in diabetic cats, which would be congruent with human studies [39,40,41].

The role of pancreatic endocrine secretions in glycemic control has been well characterized; however, the regulation of these secretions by other hormones and substrates in cats remains poorly understood. In the pancreas, we found a decrease in insulin mRNA abundance in both untreated and treated diabetic cats, which was consistent with previous reports on reductions in β-cells or insulin immunopositivity in the pancreas of diabetic cats [42,43,44,45]. This decrease in insulin mRNA could be due to toxicity of beta cells in the face of hyperglycemia. Despite the well-known effects of insulin on glucose clearance by peripheral tissues, little is known of autocrine regulation of pancreatic insulin in cats. We found a reduction in the transcript abundance of insulin receptor in untreated diabetic cats, a reduction in IRS-1 mRNA abundance in untreated diabetic and overweight cats, and an increase in PI3K transcript in treated diabetic cats; however, the protein abundances of these markers did not differ among groups. This discrepancy between mRNA and protein levels suggests potential post-transcriptional regulatory mechanisms or compensatory processes that maintain the protein levels of these signaling intermediaries to maintain insulin sensitivity in the pancreas to offset for reduced insulin biosynthesis. Given that the MAPK pathway is involved in inflammation, stress, and tumorigenesis [46], the significant decrease in MAPK-p42/44 protein abundance in the treated diabetic group is supportive of the protective effects of exogenous insulin on the pancreas and a possible reduction in the glucotoxicity of the beta cells. Previous studies have shown that phosphorylation of AKT is a critical regulatory step that facilitates glucose uptake, metabolic homeostasis, and β-cell survival [47,48,49]. In agreement with these studies, we found increased pancreatic protein levels of total AKT, phospho-AKT, and phospho-AKT/total AKT ratio in treated diabetic cats. Together, these findings support that insulin therapy in treated diabetics enhances the regulatory cascade downstream of the insulin receptor to activate AKT and that could potentially rescue β-cell functionality.

The reductions in GLUT-1 and GLUT-2 mRNA levels in the pancreas of both diabetic groups might suggest impaired glucose uptake similar to the findings of reduced glucose uptake in the pancreas of diabetic rodents [50,51,52]. However, the observed increase in GLUT-1 protein abundance in treated diabetic cats, and the tendency for increased GLUT-2 protein abundance in overweight cats, indicates potential post-transcriptional regulatory mechanisms or compensatory processes that influence the protein levels and fluxes of the transporters. Interestingly, of the incretin receptors, we found that GLP-1 receptor mRNA abundance was lower in treated diabetic than in lean cats and that the GIP receptor mRNA levels remained unchanged, whereas the protein abundance of both GLP-1 and GIP receptors were increased in treated diabetics, indicating the involvement of either a post-transcriptional regulatory or a compensatory response. We postulate that GLUT-1, GLUT-2, or GLP-1 receptor expression may be tightly regulated at the protein level to respond to immediate physiological needs, whereas mRNA levels could represent a preparatory or delayed response. Therefore, the weak incretin response in cats [34,35], greater circulating GLP-1 and GIP concentrations in diabetic cats [37], and the greater GLP-1, GIP, and GLUT1 receptor protein abundance in the pancreas of treated diabetics together indicate that exogenous insulin likely recruited incretins to protect against pancreatic decompensation in this particular cohort of diabetic cats. Interestingly, the higher plasma concentrations of incretins [37] and greater protein abundance of GLP-1 receptor in untreated diabetics together with the lower pancreatic insulin transcript abundance suggest that feline diabetes is unlikely to be due to a deficit in incretin secretion as in human diabetes [24,53], but very likely due to impaired incretin receptor signaling to potentiate insulin secretion from the pancreas.

We evaluated key markers of insulin signaling in skeletal muscles in our population, as skeletal muscle contributes to over 85% of glucose disposal in humans of normal weight [54], and deficits in proximal insulin receptor signaling are associated with diabetes [55]. We observed a significant decrease in insulin receptor mRNA abundance in the muscle of untreated diabetic cats, whereas the mRNA abundance of the downstream regulator PI3K was decreased and MAPK3 mRNA levels were increased in the muscle of overweight and diabetic cats. The downregulation of muscle PI3K transcript, but unaltered IRS-1 transcript, in the overweight cats was in agreement with another study on diet-induced obesity in cats [18]. At the protein level, insulin receptor abundance remained unchanged across groups, whereas PI3K-p85α protein abundance tended to decrease in treated diabetic cats. These findings suggest that proximal insulin signaling in the muscle is marginally affected in diabetic cats. A previous study reported a reduction in GLUT-4 protein in the muscle of obese cats [27]. We expand on this finding and now demonstrate a significant reduction in muscle GLUT-4 protein abundance across all diabetic and overweight groups, which highlights a critical impairment in glucose transport capacity. GLUT-4 is a key mediator of insulin-stimulated glucose uptake in skeletal muscle, and its reduction is indicative of the profound impact of impaired muscle glucose uptake in diabetes [56]. Regarding incretin signaling, the muscle GLP-1 receptor protein abundance decreased in untreated diabetic cats and tended to decrease in overweight cats. This reduction in GLP-1 receptor expression aligns with impaired incretin signaling in the skeletal muscle in cats and may contribute to reduced insulin sensitivity as reported in rodents [57]. Moreover, the reduction in MAPK-p42/44 protein levels in untreated diabetic cats further supports the notion of disrupted insulin signaling in the skeletal muscle. MAPK signaling is involved in a range of cellular processes, including stress response and growth regulation [58,59], and its decline may reflect a loss of protective cellular adaptations in untreated diabetes. Given the increased lipid content in the skeletal muscle of overweight and diabetic cats, it is also likely that lipotoxicity may contribute to insulin resistance in skeletal muscle, consistently with other species [19,60,61]. Despite these caveats, our findings suggest that impaired insulin and incretin signaling may contribute to insulin resistance in skeletal muscle at least at the transcript level by interfering with insulin signaling downstream of the insulin receptor and are in general consistent with previous studies in diabetic humans [62].

Second to muscle, the liver contributes to over 5% of glucose disposal in normal weight humans [55], with enhanced hepatic glucose production likely due to impaired insulin signaling in diabetic humans [55,63]. We found that in the liver, insulin receptor mRNA abundance tended to increase in treated diabetic compared with lean cats, which is supportive of enhanced response to exogenous insulin in diabetics. However, insulin receptor protein levels did not differ among groups, a phenomenon suggestive of post-transcriptional regulation that might imply poor glucose control in this particular cohort of treated diabetic cats. The upregulation of GLP-1 receptor transcripts with no significant changes in protein abundance in untreated diabetics is suggestive of compensatory upregulation of incretin signaling at the transcript level in the liver to counteract insulin resistance, and the downregulation of GLUT-1 and GLUT-2 transcripts in diabetics is suggestive of impaired glucose uptake in the liver. Interestingly, we observed decreased protein abundance of PI3K-p85α and increased protein abundance of total AKT in overweight cats. Previous studies have shown that PI3K-p85α transcript is reduced in the liver of diet-induced obese cats [18] and that PI3K-p85α is a negative regulator of insulin signaling, with PI3K-p85α deficient mice having increased insulin sensitivity [64,65]. AKT, a downstream target of PI3K, appears to be essential for insulin signaling, as AKT-deficient mice have impaired insulin-induced glucose uptake in the liver and muscle [66,67], and humans with partial-loss-of function mutations in AKT2 have reduced glucose uptake in the liver and muscle and increased risk of diabetes [68]. Hence, the reciprocal reduction in PI3K-p85α and increase in AKT in the liver might partly contribute to the maintenance of euglycemia in our overweight cats. Despite the lack of differences in IRS-1, PI3K-p85α, and AKT, we observed increased protein abundance of MAPK-p42/44 in untreated diabetic cats. As MAPK-p42/44 is one of the key markers that cause hepatic inflammation [69], our findings suggest potential activation of inflammatory and stress pathways that might contribute to the pathophysiology of diabetes in cats.

Potential limitations of our study include the inability to accurately assess total body adiposity with MRI or DEXA scans; lack of complete dietary histories; variability in the duration of diabetic status before presentation and euthanasia; absence of histopathological assessments in peripheral tissues; and lack of measurements of serum concentrations of insulin, GLP-1, and GIP, as standardization of last meal fed was not considered possible in our population. Assessment of insulin sensitivity would have been of interest to further the understanding of our findings; however, it was not possible in clinical patients. As feline DM is diagnosed according to ALIVE criteria [70], because of later presentation of disease compared with humans, insulin sensitivity tests are not standard practice to perform in cats. We cannot exclude that other factors were impacting insulin signaling and receptor activity; however, we attempted to exclude cases with chronic illnesses that may have influenced incretin hormone release, insulin, or incretin signaling in organs of interest or significant disease or therapy that may have led to insulin resistance. We focused on key regulatory molecules of incretin and insulin signaling in target tissues by quantifying the transcript and relative protein abundance but did not assess the biological activities of the molecules. A strength of the study is the recruitment of patients with naturally occurring diabetes and overweight status. 

In summary, our study provides important insights into the complex mechanisms by which insulin and incretin signaling potentially regulate glucose homeostasis in cats. Our findings suggest that, similarly to human diabetes, ectopic lipid deposition in the liver and skeletal muscle, impaired incretin signaling in the pancreas, and dysfunctional hepatic insulin signaling may together contribute to insulin resistance and the development of diabetes in cats. Larger studies could be performed to glean further understanding of sex differences in insulin sensitivity and signaling in the lean, overweight, and diabetic groups. The development of pharmacological and dietary approaches that could correct these deficits may lead to new avenues for preventing and treating feline diabetes, with potential translational significance for humans. Importantly, given that the study population was client-owned pets, the outcomes from the current study have broader one-health significance and might lead to a deeper understanding of the pathophysiological mechanisms of human obesity and diabetes using naturally occurring feline models that might better simulate the human conditions. 

## 4. Materials and Methods

### 4.1. Animals and Sample Collection

The study received approval from the University of Calgary Veterinary Science Animal Care Committee (AC16-0034/AC20-0023) and the Western College of Veterinary Medicine Animal Research Ethics Board (20160052/20200051), and all experiments were performed in accordance with relevant guidelines and regulations. In a prospective cross-sectional study design, a cohort of cats was recruited between May 2016 and February 2020 in the Calgary and Saskatoon regions, Canada, involving local veterinarians who learned of the study through direct communication, emails, word of mouth, and posters provided to participating hospitals. When veterinarians identified suitable cases that were undergoing euthanasia for reasons unrelated to the study, participation in procuring blood and tissue samples was discussed with pet owners. The researchers CJM and ECS were then contacted to ensure that pets were suitable, and arrangements were made to collect samples within 20 min of euthanasia. Before euthanasia, informed consent for tissue and blood collection was obtained from pet owners. The animals were euthanized with intravenous injection of pentobarbital sodium (Euthanyl^®^, MTC Animal Health Inc., Cambridge, ON, Canada) following standard dosing guidelines from the American Veterinary Medical Association [71]. Exclusion criteria for the study included history of chronic enteropathies, suspicion of acute pancreatitis, gross morphological changes identified when procuring tissues of the organs of interest (mass lesions), therapy with corticosteroids, or hyperthyroidism. Inclusion criteria were lean, overweight or obese, and diabetic cats at or above 7 years of age; this age was chosen to best represent a population of diabetics. Diabetic status was diagnosed based on appropriate number of ALIVE criteria to include compatible clinical signs of hyperglycemia, glucosuria, and increased glycated protein [70]. Nondiabetic cats meeting inclusion criteria were categorized based on body condition scores (BCS) using a 9-point system [72] as either lean (BCS ≤ 5) or overweight or obese (BCS ≥ 6). A total of 67 cats were recruited, and their tissues were sampled. The cats were classified into three groups: lean (LC; *n* = 22), overweight/obese (OW; *n* = 17), or diabetic (DC; *n* = 29). 

A total of 29 diabetic cats (DC) at 5–18 years of age were recruited, and of these, 3 were excluded because of the presence of a pancreatic mass when procuring tissues, 1 was excluded because of low fructosamine and blood glucose at the time of euthanasia, and 1 was excluded for hyperthyroidism. After exclusion criteria were applied, this left 24 cats for further analysis. The diabetic group (*n* = 24) was further stratified based on whether cats received exogenous insulin treatment (TD; *n* = 8) [45] or were untreated (UD; *n* = 16). Cats defined as treated were receiving exogenous insulin daily for management. Untreated cats were defined as those that had not received daily insulin for more than 2 weeks. Only one UD was treated with insulin. This case was first diagnosed as diabetic while presenting with diabetic ketoacidosis and was started on regular insulin; however, it was euthanized within 24 h. Reasons for euthanasia in the TD were hypoglycemic events (2), trauma, acute onset seizure, behavioral (2), and inability to continue treatment. The remaining cases were euthanized because of treatment not being pursued by the owners of the patients because of the diabetic state itself. After exclusions, the UD group had 8 neutered males and 8 spayed females, with 12 domestic short-hair and 4 domestic long-hair, and the TD group had 4 neutered males and 4 spayed females, with 6 domestic short-hair, 1 domestic long-hair, and 1 Himalayan breed. For the TD group, cats included were treated with exogenous insulin from 3 weeks to 5 years, and of these, 5 were treated with Lantus^®^ (Sanofi- Aventis Canada Inc., Toronto, ON, Canada) subcutaneously every 12 h, 2 with ProZinc^®^ (Boehringer Ingelheim Animal Health Canada Inc., Burlington, ON, Canada) subcutaneously every 12 h, and 1 with Caninsulin^®^ (Merck animal Health. Intervet Canada Corp., Kirkland, QC, Canada) subcutaneously every 12 h. The diabetics were an independent group and were not part of the lean or overweight groups. The diabetic cats had BCS performed; however, they were not further subdivided as lean or overweight for analysis based on BCS. Diet was not consistently reported.

A total of 22 lean cats (LC) at 7–22 years of age were recruited, and of these, 7 were excluded for hyperthyroidism (4), hepatopathy (1), and concerns for significant comorbid conditions due to low body condition (2). Reasons for euthanasia for cases included for tissue analysis included acute renal injury, anemia (2), musculoskeletal disease, respiratory disease, dental disease (3), and behavioral issues (5). After exclusions, the LC group had 8 neutered males and 7 spayed females, with 14 domestic short-hair and 1 Himalayan breed. 

A total of 17 overweight cats (OC) at 7–17 years of age were recruited, and 2 were excluded based on the presence of elevated thyroid hormone concentrations. Reasons for euthanasia in this group undergoing tissue analysis included anemia, pulmonary mass, subcutaneous mass, acute vomiting, dental disease (2), musculoskeletal disease, neurologic disease, and behavioral concerns (7). Following exclusions, the OC group had 7 neutered males and 8 spayed females, with 11 domestic short-hair and 4 domestic long-hair cats.

Morphometric measurements, including body mass index (BMI) and body fat percentage, were assessed using tape measures and digital scales since more advanced techniques such as DEXA or MRI were not available or feasible. The detailed equation for BMI was BMI = body weight (kg)/(body length [meters]) × height [meters]), as previously described [73,74]. Further, body fat percentage was calculated as body fat % = [([RC/0.7062] − LIM)/0.91560) − LIM], where RC was the circumference of the rib cage [28] and LIM was the length of the lower limb from the middle of the patella to the dorsal hip [28].

Physical examination was performed premortem by the primary care veterinarian or the investigators (CJM or ECS). Blood was drawn at the time of catheter placement for euthanasia whenever possible to limit patient stress, or by venipuncture if necessary. Blood samples were collected, packed cell volume measured, and serum separated and sent for biochemical profile, which included serum glucose, Total T4, and fructosamine (Antech Diagnostics, Calgary, AB, Canada). If these analyses were already performed on a patient within 7 days prior to euthanasia, no additional blood samples were collected. Urine was collected from the bladder during tissue sampling, and a USG and urine dipstick analysis was performed. While procuring tissues, ECS and CJM grossly assessed the pancreas, liver, and small and large intestines. Samples of the pancreas were obtained in a standardized manner from the left limb, and skeletal muscle was obtained from the rectus abdominus parallel to the midline incision. Pancreatic, skeletal muscle, and liver samples were collected, wrapped in aluminum foil, placed in liquid nitrogen, and then stored at −80 °C for later tissue analyses. Medical histories were obtained from records and discussions with veterinarians to minimize owner impacts during euthanasia. Some cats had incomplete historical information if not with the original owners.

### 4.2. Tissue Composition

Liver and muscle biopsy samples were weighed (~100 mg) from all groups. Fat mass content was measured in duplicate by combined NMR T1 and T2 relaxation measurements and chemometric data analysis, with a noninvasive quantitative magnetic resonance method using the tissue biopsy probe of the Minispec LF-110 NMR analyzer (Bruker Corp., Milton, ON, Canada) following our previously published procedures [75]. 

### 4.3. Tissue RNA Isolation and qPCR

The general procedures for RNA isolation using RNeasy Mini Kit (Qiagen Inc., Toronto, ON, Canada) and RT-qPCR were according to our published procedure [76,77]. Briefly, pancreas, liver, and muscle samples were placed in 2 mL sterile polypropylene microvials (Cat# 522s, Biospec Products, Inc., Bartlesville, OK, USA) with addition of 1 mL QIAzol lysis reagent (Qiagen, ON, Canada) and 1 mm diameter glass beads (Cat# 11079110, Biospec Products, Bartlesville, OK, USA), followed by homogenization using a bead beater (Mini-Beadbeater-16, Cat# 607, Biospec Products, Bartlesville, OK, USA) for 3 min. After carefully piercing the fat layer with a Pasteur pipette, 200 µL chloroform was added to each sample and centrifuged at 10,700 rpm for 15 min. Next, 600 µL of 70% ethanol was added, and samples were briefly vortexed to precipitate the RNA. Later, samples were filtered and washed with wash buffers (RW1 and RPE) to remove impurities, and RNA was eluted with 40 µL of preheated (70 °C) RNAse-free water. The RNA concentrations were measured using a NanoDrop ND-1000 spectrophotometer (Thermo Fisher Scientific Inc., Mississauga, ON, Canada), and sample concentrations were standardized to 250 ng/µL. Next, the samples were treated with amplification grade deoxyribonuclease-I (Invitrogen, Burlington, ON, Canada) to eliminate DNA, and complimentary DNA (cDNA) was synthesized using 250 ng/µL RNA and 5× RT Buffer, Superscript II, RNase OUT Recombinant Ribonuclease Inhibitor, and dNTPs-100µM (Life Technologies, Carlsbad, CA, USA) on a Mastercycler Pro thermocycler (Eppendorf Canada Ltd., Mississauga, ON, Canada). The following program was used for cDNA synthesis: 22 °C for 5 min, followed by 42 °C for 30 min, 85 °C for 5 min, and terminated at 4 °C. qPCR analyses were performed on a Mastercycler EP Realplex thermocycler (Eppendorf Canada Ltd.) with 12.5 µL Power SYBR Green PCR Mix (Applied Biosystems Inc., Waltham, MA, USA), 100 µM of primers, and 2 µL of cDNA in a 25 µL reaction in duplicates. Cycle condition was as follows: denaturation: 50 °C for 2 min and 95 °C for 10 min, 40 cycles amplification: 95 °C for 15 s and 60 °C for 1 min, followed by a melt curve program: 95 °C for 15 s, 60 °C for 1 min, and 95 °C for 15 s. Primers for qRT-PCR targeting key markers of glucose regulation, insulin signaling, and incretin signaling are presented in Supplementary Table S1. Ribosomal protein S7 (RPS7) served as internal control. Relative differences in the expression of target genes were determined using the 2^−∆∆CT^ method [78,79].

### 4.4. Protein Isolation and Immunoblotting

Pancreas, liver, and muscle samples (100–200 mg) from cats were each placed in 2 mL sterile microvials (Cat# 522s, Biospec Products Inc., Bartlesville, OK, USA) along with 1 mm diameter glass beads (Cat# 11079110, Biospec Products Inc., Bartlesville, OK, USA). Samples were homogenized in 660 μL of a mix of NP40 buffer (Invitrogen, Burlington, ON, Canada), 10× protease inhibitor cocktail, and 0.3 M phenylmethanesulfonyl fluoride (Sigma-Aldrich, Oakville, ON, Canada). The cellular debris was removed by centrifuging the homogenates at 2500× *g* for 15 min (4 °C). Cell lysate was carefully decanted, and protein concentrations were determined using the Bradford assay (Bio-Rad Lab, Mississauga, ON, Canada). Pancreas and liver samples were diluted to 2 mg/mL, and muscle samples were diluted to 20 mg/mL, for immunoblot. Briefly, protein extracts were diluted to 2 mg/mL and loaded on either a 4–20% Mini-Protean TGX stain-free gel (BioRad Cat# 4568094) or 10% Tris-glycine SDS-polyacrylamide gel (1.5 M Tris-pH 8.8, 30% acrylamide, 10% sodium dodecyl sulfate and ammonium persulfate), resolved for 1–2 h at 90–100 V (50 mM Tris base, 50 mM glycine, 0.05% SDS), and were transferred to 0.2 μM nitrocellulose membranes for ~2 h at 100 V (25 mM Tris base, 192 mM glycine, 20% methanol). The blots were then blocked overnight with 5% bovine serum albumin (BSA) in TBST (50 mM Tris base, 150 mM NaCl, 0.1% Tween-20, pH 7.6) at 4 °C, incubated with primary antibodies for 4 h at 4 °C, washed thrice with TBST, and subsequently incubated with a secondary antibody conjugated with horseradish peroxidase for 1 h at 4 °C. Following the addition of luminol (Clarity Western ECL substrate, Bio-Rad Laboratories Ltd. Cat# 1705061, Hercules, CA, USA), proteins were detected and quantified using either an ChemiDoc MP imaging system (Bio-Rad Laboratories Ltd., Mississauga, ON, Canada) or an Azure 600 (Azure Biosystems, Dublin, CA, USA). The band intensity of each target protein was normalized to the internal loading controls for assessing the relative amount of protein content within the sample. The internal loading controls β-actin (42 kDa) and HSP70 (~70 kDa) for each marker were selected to ensure adequate resolution of the bands between the loading control and target protein. The dilutions of primary and secondary antibodies are provided in Supplementary Table S2. Prior to quantifying the relative abundance of markers related to insulin signaling and lipid metabolism, a dose–response was performed using 0, 0.46, 0.93, 1.87, 3.75, 7.5, 15, and 30 mg/mL of pooled protein samples from the pancreas (Supplementary Figure S1) and liver (Supplementary Figure S2) of lean, overweight, and untreated and treated diabetic cats.

### 4.5. Statistical Analysis

Differences from the lean group in tissue composition and transcript and protein abundance were checked for normality, and non-normal data were log-transformed followed by one-way ANOVA with Benjamini–Hochberg corrections for multiple comparisons with a false discovery rate of 0.05 [80] using GraphPad Prism 10.1^®^. Data were expressed as mean ± SEM, and significance was set at *p* ≤ 0.05.

## Figures and Tables

**Table 1:** Body and tissue measurements in lean, overweight, and untreated and treated diabetic cats. Measurements include body weight (BW, kg), body fat percentage, body mass index (BMI), body condition score (BCS), blood glucose (mmol/L) and fructosamine (µmol/L), and percentages of liver fat and muscle fat. Data were analyzed by one-way ANOVA, as appropriate, with Benjamini–Hochberg corrections for multiple comparisons and a false discovery rate of 0.05. Values are mean ± SEM, ^a^ *p* < 0.05 lean vs. overweight, ^b^ *p* < 0.05 lean vs. untreated diabetic, ^c^ *p* < 0.05 lean vs. treated Diabetic, * *p* < 0.10 lean vs. treated Diabetic.

	Lean	Overweight	Untreated Diabetic	Treated Diabetic
Age (yrs)	10.43 ± 0.91	11.87 ± 0.72	10.47 ± 0.95	12.06 ± 0.21
Sex				
Male	8	7	8	4
Female	7	8	8	5
Body weight (kg)	3.88 ± 0.25	6.16 ± 0.45 ^a^	5.03 ± 0.34 ^b^	5.27 ± 0.68 ^c^
	(*n* = 14)	(*n* = 11)	(*n* = 14)	(*n* = 5)
Body fat (%)	26.74 ± 1.57	41.25 ± 3.13 ^a^	30.49 ± 1.32	34.94 ± 6.09 *
	(*n* = 14)	(*n* = 11)	(*n* = 14)	(*n* = 5)
Body mass index	2.62 ± 0.24	3.49 ± 0.21 ^a^	2.91 ± 0.19	3.30 ± 0.52
	(*n* = 14)	(*n* = 11)	(*n* = 14)	(*n* = 5)
Body condition score	4.57 ± 0.17	7.18 ± 0.33 ^a^	5.50 ± 0.33 ^b^	6.40 ± 0.81 ^c^
	(*n* = 14)	(*n* = 11)	(*n* = 14)	(*n* = 5)
Glucose (mmol/L)	4.98 ± 0.31	7.19 ± 0.51	22.27 ± 1.31 ^b^	20.56 ± 6.79 ^c^
	(*n* = 14)	(*n* = 11)	(*n* = 14)	(*n* = 5)
Fructosamine (μmol/L)	245.54 ± 11.62	240 ± 7.49	540.29 ± 33.41 ^b^	481.20 ± 79.46 ^c^
	(*n* = 13)	(*n* = 11)	(*n* = 14)	(*n* = 5)
Liver fat (%)	21.77 ± 0.91	21.25 ± 0.69	28.73 ± 1.82 ^b^	24.35 ± 3.21
	(*n* = 16)	(*n* = 13)	(*n* = 17)	(*n* = 7)
Muscle fat (%)	7.94 ± 1.04	16.13 ± 3.24 ^a^	9.53 ± 1.20	18.92 ± 5.40 ^c^
	(*n* = 13)	(*n* = 16)	(*n* = 17)	(*n* = 7)

## Data Availability

The raw data supporting the conclusions of this article will be made available by the authors on request.

## Supplementary Materials

The following supporting information can be downloaded at: https://www.mdpi.com/article/10.3390/ijms252313195/s1.
