# Supplementary material for: Feline Diabetes Is Associated with Deficits in Markers of Insulin Signaling in Peripheral Tissues
Source: Int J Mol Sci. 2024 Dec 8;25(23):13195. doi: 10.3390/ijms252313195 (PMC11642086; doi:10.3390/ijms252313195)

Supplementary Information

Feline diabetes is associated with deficits in markers of insulin signaling in peripheral tissues.

Souvik Patra[1], Chantal J. McMillan[2]*, Elisabeth R. Snead[3], Amy L. Warren[2], Kevin Cosford[3], and Prasanth K. Chelikani[1,2]*

[1]School of Veterinary Medicine, Texas Tech University, 7671 Evans Drive, Amarillo, Texas, TX 79106, USA.

[2]Faculty of Veterinary Medicine, University of Calgary, 3280 Hospital Dr. NW, Calgary, AB, Canada.

[3]Department of Small Animal Clinical Sciences, Western College of Veterinary Medicine, University of Saskatchewan, Saskatoon, Canada.

***Corresponding Authors**

Chantal J. McMillan, DVM, MVSc, DACVIM, Faculty of Veterinary Medicine, University of Calgary, 3280 Hospital Dr. NW, Calgary, AB, Canada. E-mail: cjmcmill@ucalgary.ca, phone: +1 (403) 210-6002.

Prasanth K. Chelikani, BVSc, MVSc, PhD, FTOS, School of Veterinary Medicine, Texas Tech University, 7671 Evans Drive, Amarillo, TX 79106, USA, E-mail: pchelika@ttu.edu, phone: +1 (806) 834-5697

Supplementary Information

Supplementary Table S1. The primer sequence (forward, F, and reverse, R), location on template (base pairs, bp), amplicon size, and GenBank accession numbers for target and reference genes used for qPCR in the current study.

Target gene	Forward sequence (5'-3')	Reverse sequence (3'-5')	Product size (bp)	Annealing temperature (°C)	GenBank Accession Number/Source
RPS7	GTCCCAGAAGCCGCACTTT	CACAATCTCGCTCGGGAAAA	74	64	NM_001009832.1
Insulin	TTCGTCAACCAGCACCTGTG	CACAGCATTGCTCCACGATG	217	60	DOI:10.1016/j.tvjl.2008.11.012
Insulin Receptor	CTGCCCTGCCACTGTCATC	GCAGACGGTCGGACAAACTT	85	60	DQ835565
IRS-1	ACCTGCGTTCAAGGAGGTCTG	CGGTAGATGCCAATCAGGTTC	81	60	AB436530
IRS-2	TGGCAGGTGAACCTGAAGC	GAAGAAGAAGCTGTCCGAGTGG	177	60	AB436531
GLUT-1	ATTGTGGCTGAACTCTTCAG	CCAGGAGTACGGTGAAGATG	157	60	DQ640900
GLUT-2	GCGCCCTTGGCACACTT	AGGCCGACAATCTGACTAATGAG	67	60	DOI: 10.1016/j.tvjl.2008.11.012
PI3K	GCATTAAACCAGACCTCATTCAGC	GCGAGTATTGGTCTTCAGTGTTCTC	132	60	AB436617
MAPK3	GGCCCGAAACTACCTACAGT	AGCTCCATGTCGAAGGTGAA	228	60	XM_023247125.1
GLP1-R	CCTCCTGAGCTTCAGACACC	CTTGACAATGCCCCAAGGGA	370	61.8	XM_019831583.1
GIPR	GAGGCTCTGTGGGGCAGA	CAGCCGCCTGAAGAAACTCA	422	60	XM_011289805.1
ACC	CGCCAGGTTCTTATTGCCTC	TCCTCACTACCTGGTTGCTG	196	60	XM_019817557.2
FAS	GGAAGTTGTGATTGCTGGCA	AAGGAGGCATCGAACTTGGA	186	60	XM_023244367.1

Supplementary Information

Supplementary Table S2. The primary and secondary antibodies, antibody type, source company, catalog number, and working dilution of antibodies used for immunoblotting in the current study.

**Primary antibody**	**Type**	**Company**	**Catalogue number**	**Working dilution**
Beta Actin	Mouse monoclonal	Sigma	A5441	1:5000
HSP70	Mouse monoclonal IgG	Sigma	H5147	1:5000
Insulin	Mouse monoclonal IgG	Sigma	I2018	1:1000
Insulin receptor	Rabbit monoclonal IgG	Cell Signaling	3025	1:1000
PI3K-p85α	Mouse monoclonal IgG	Santa Cruz	Sc-1637	1:200
MAPK3 (p42/44, Thr202/Tyr204)	Rabbit polyclonal	Cell Signaling	9101	1:1000
Total AKT	Rabbit polyclonal IgG	Cell Signaling	9272S	1:1000
Phospho-AKT	Mouse monoclonal IgG	Cell Signaling	4051S	1:1000
IRS-1	Rabbit polyclonal IgG	Cell signaling	2382	1:1000
GLUT-1	Rabbit monoclonal	Cell Signaling	12939	1:1000
GLUT-2	Rabbit polyclonal IgG	Milipore- Sigma	07-1402-I	1:1000
GLUT4	Mouse monoclonal IgG	Cell Signaling	2213	1:1000
GLP-1 receptor	Mouse monoclonal IgG	Santa Cruz	Sc-390774	1:100
GIPR	Rabbit polyclonal IgG	Thermo-Invitrogen	PA5-14408	1:1000
ACC	Rabbit polyclonal IgG	Cell Signaling	3662	1:1000
Phospho-ACC (Ser79)	Rabbit polyclonal IgG	Cell Signaling	3661	1:1000
FASN	Rabbit monoclonal IgG	Cell Signaling	3180S	1:1000
HSL	Rabbit polyclonal IgG	Thermo Fisher	PA1-16966	1:100
Phospho-HSL (Ser660)	Rabbit polyclonal IgG	Thermo Fisher	PA5-64494	1:1000

**Secondary antibody**	**Type**	**Company**	**Catalogue number**	**Working dilution**

Goat anti-mouse	IgG HRP	Promega	W4021	1:2500
Goat anti-rabbit	IgG HRP	Promega	W4011	1:2500

Supplementary Information

Supplementary Table S3. The sample size (n) for each molecular target and treatment group that are reported in Figs 1-3.

Target	Lean (n)	Overweight (n)	Untreated Diabetic (n)	Treated Diabetic (n)
Pancreas Insulin mRNA	15	15	16	7
Pancreas Insulin receptor mRNA	14	15	16	8
Pancreas IRS-1 mRNA	14	14	16	7
Pancreas IRS-2 mRNA	14	14	15	7
Pancreas MAPK mRNA	14	15	15	8
Pancreas PI3K mRNA	13	15	12	8
Pancreas GLUT1 mRNA	13	15	16	7
Pancreas GLUT2 mRNA	15	15	16	7
Pancreas GLP1R mRNA	14	14	12	8
Pancreas GIPR mRNA	11	8	8	3
Pancreas Insulin receptor	13	14	14	7
Pancreas IRS-1 protein	5	5	5	5
Pancreas PI3K protein	8	12	13	4
Pancreas MAPK-p42/44 protein	13	14	14	5
Pancreas AKT protein	13	13	15	7
Pancreas phospho-AKT protein	11	15	15	6
Pancreas GLUT- 1 protein	10	12	13	5
Pancreas GLUT- 2 protein	12	14	15	5
Pancreas GLP-1 receptor protein	12	13	16	6
Pancreas GIP receptor protein	12	15	16	7

Muscle Insulin receptor mRNA	15	14	14	9
Muscle IRS-1 mRNA	14	14	14	9
Muscle MAPK mRNA	14	14	14	9
Muscle PI3K mRNA	12	14	14	9
Muscle GLP-1 receptor protein	14	12	14	7
Muscle GLUT-4 receptor protein	12	12	15	7
Muscle Insulin receptor protein	13	12	15	7
Muscle PI3K-p85α protein	13	12	15	6
Muscle MAPK-p42/44 protein	12	12	14	7
Liver Insulin receptor mRNA	14	13	16	7
Liver GLUT1 mRNA	15	13	15	6
Liver GLUT2 mRNA	14	13	16	6
Liver GLP-1 receptor mRNA	13	12	13	6
Liver ACC mRNA	15	13	17	6
Liver FAS mRNA	15	12	12	7
Liver Insulin receptor protein	13	12	15	7
Liver GLP-1 receptor protein	15	13	14	5
Liver IRS-1 protein	13	11	14	6
Liver PI3K protein	12	11	11	7
Liver MAPK protein	12	10	13	6
Liver AKT protein	11	14	12	7
Liver ACC protein	14	12	10	6
Liver phospho-ACC protein	13	10	15	6

Liver FAS protein	15	14	14	7
Liver HSL protein	15	13	13	6
Liver phospho-HSL protein	14	13	13	6

Supplementary Information

Supplementary Figure S1. Immunoblot dose-response of pancreas insulin signaling, glucose regulation and incretin signaling markers. Protein abundance of **(a)** beta actin, **(b)** insulin, **(c)** insulin receptor, **(d)** insulin receptor substrate (IRS)-1, **(e)** phosphoinositide 3-kinase (PI3K), **(f)** glucose transporter (GLUT)-1, **(g)** glucose transporter (GLUT)-2, and **(h)** glucagon like peptide (GLP)-1 receptor at loading concentrations of 0, 0.46, 0.93, 1.87, 3.75, 7.5, 15 and 30 mg/mL/well, respectively.

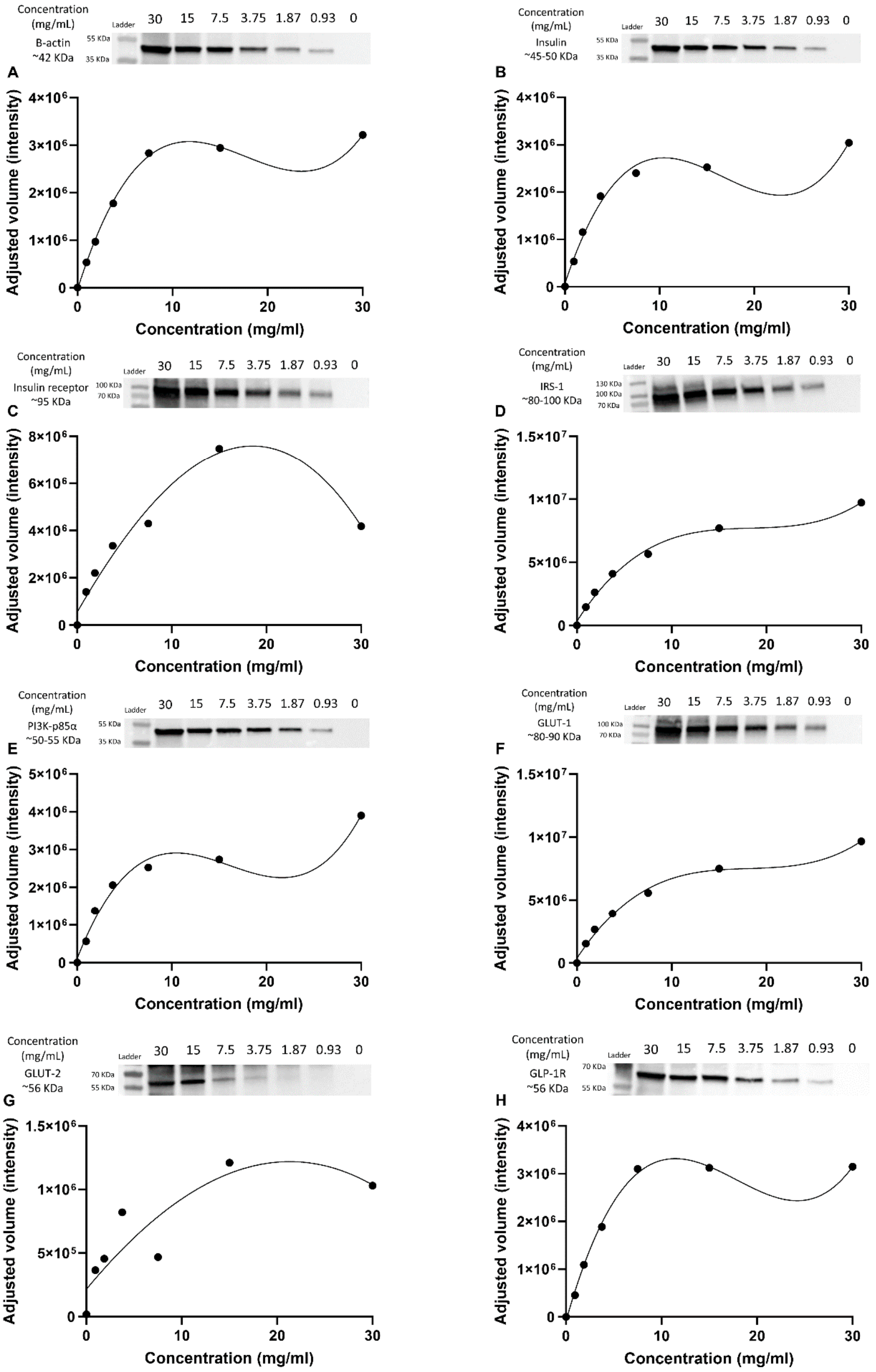

Supplementary Figure S2. Immunoblot dose-response of liver insulin signaling markers. Protein abundance of **(a)** insulin receptor, **(b)** insulin receptor substrate (IRS)-1, **(c)** phosphoinositide 3-kinase (PI3K), **(d)** mitogen activated protein kinase (MAPK)-3, **(e)** protein kinase B (AKT) at loading concentrations 0, 0.46, 0.93, 1.87, 3.75, 7.5, 15 and 30 mg/mL/well, respectively.

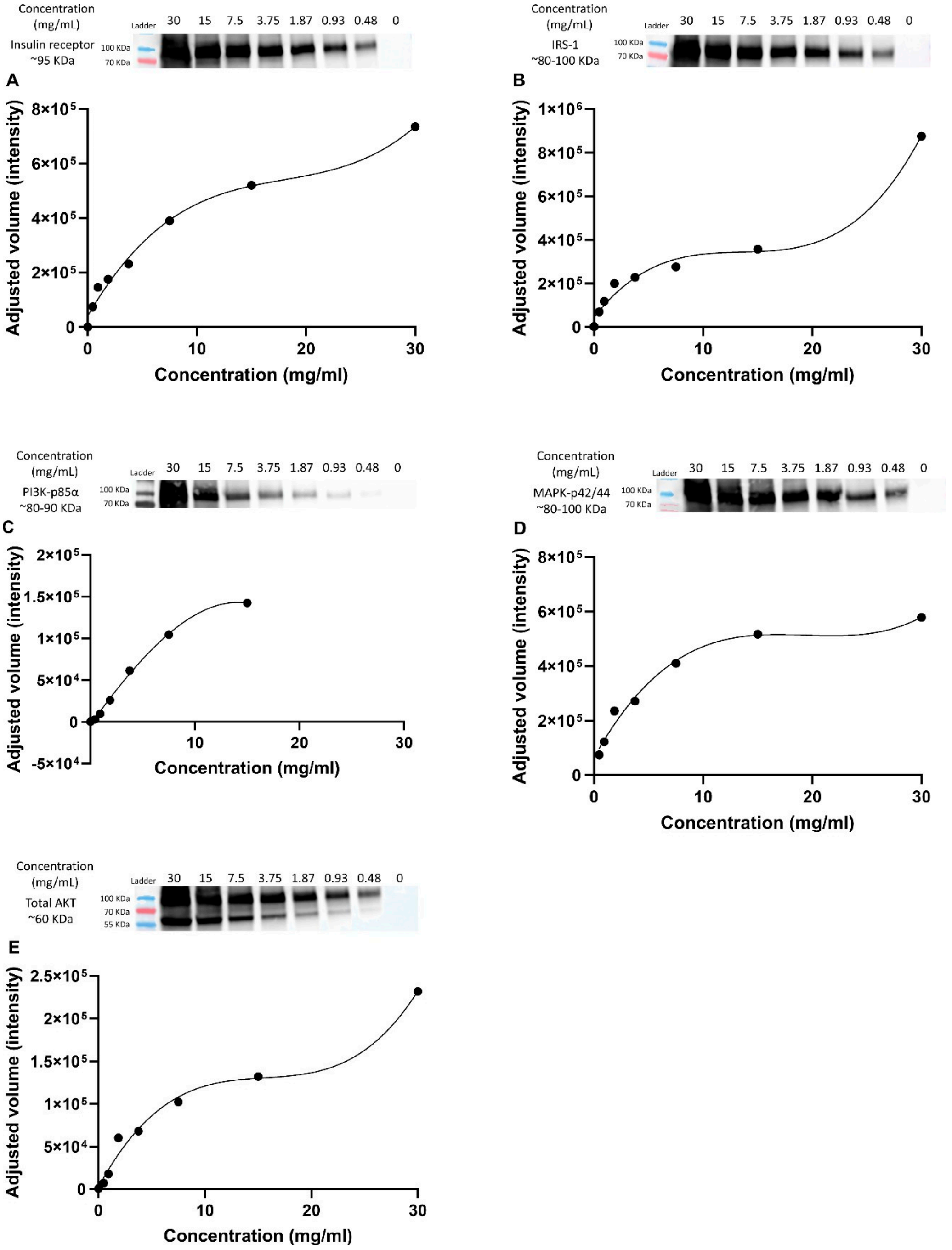

Supplementary Information

Supplementary Figure S3. The original, uncropped images of the blots correspond to Figure 1B (pancreas insulin receptor) in the manuscript. Please note that sample ID for example 1LC indicates animal ID (#1) of the lean cat group (LC). Group abbreviations are: LC=lean cat; OW=overweight; UD=untreated diabetic; TD=treated diabetic. Blots/bands highlighted in red box represent the images shown in the manuscript.

Blot 1C.1: The original, uncropped blot of pancreas insulin receptor consisting of lean (LC), overweight (OW), untreated diabetic (UD) and treated diabetic (TD) samples. (From left to right) Lane-1: ladder; Lane-2: 8LC; Lane-3: 6OW; Lane-4: 8UD; Lane-5: 21TD.

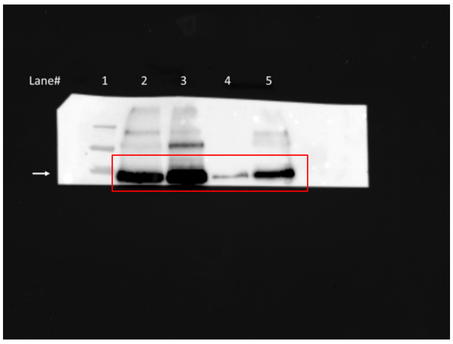

Supplementary Information

Supplementary Figure S4. Normalization control (HSP70) for pancreas insulin receptor, PI3K and MAPK in Figure 1. Blots/bands highlighted in red box represent the images shown in the manuscript.

Blot 1I.1: The original, uncropped blot of internal control (HSP70) for pancreas insulin receptor consisting lean (LC), overweight (OW), untreated diabetic (UD) and treated diabetic (TD) samples. (From left to right) Lane-1: ladder; Lane-2: 8LC; Lane-3: 6OW; Lane-4: 8UD; Lane-5: 21TD.

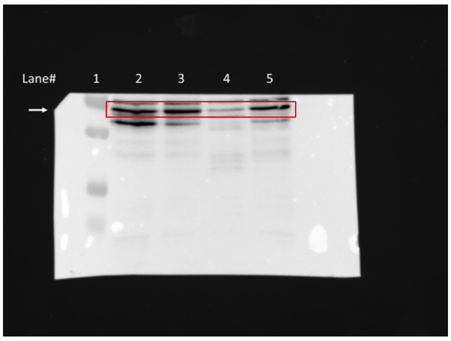

Supplementary Information

Supplementary Figure S5. The original, uncropped images of the blots correspond to Figure 1C (pancreas IRS-1) in the manuscript. Please note that sample ID for example: 1LC indicates animal ID (#1) of the lean cat group (LC). Group abbreviations are: LC=lean cat; OW=overweight; UD=untreated diabetic; TD=treated diabetic. Blots/bands highlighted in red box represent the images shown in the manuscript.

Blot 2A: (from left to right) Lane-1: ladder; Lane-2: 8OW; Lane-3: 8UD; Lane-4: 10TD; Lane-5: 15LC; Lane-6: 13OW; Lane-7: 16UD; Lane-8: 14TD; Lane-9: 16LC; Lane-10: 14OW.

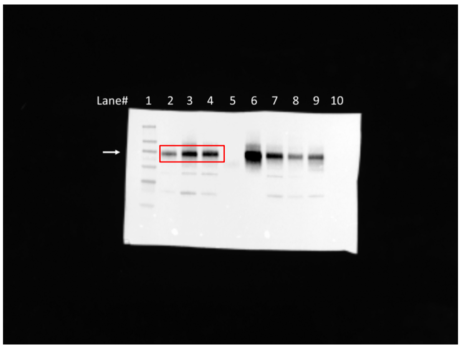

Blot 2B: (from left to right) Lane-1: ladder; Lane-2: 17UD; Lane-3: 27TD; Lane-4: 18LC; Lane-5: 17OW; Lane-6: 25UD; Lane-7: 30TD.

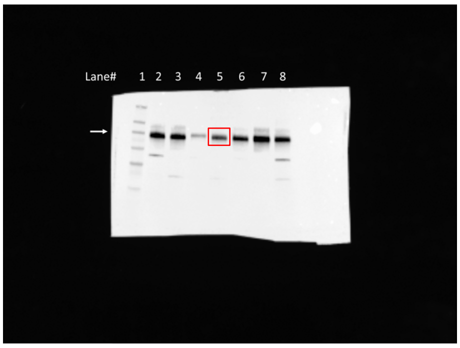

Normalization control (beta actin) for pancreas IRS-1:

Blot 2C: (from left to right) Lane-1: ladder; Lane-2: 8OW; Lane-3: 8UD; Lane-4: 10TD; Lane-5: 15LC; Lane-6: 13OW; Lane-7: 16UD; Lane-8: 14TD; Lane-9: 16LC; Lane-10: 14OW.

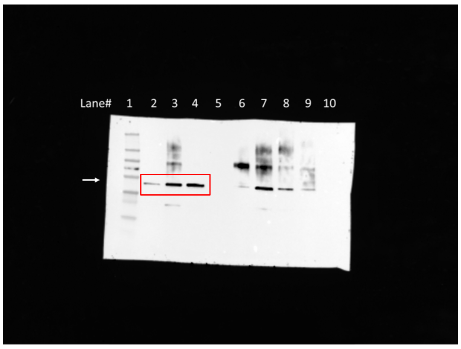

Supplementary Information

Blot 2D: (from left to right) Lane-1: ladder; Lane-2: 17UD; Lane-3: 27TD; Lane-4: 18LC; Lane-5: 17OW; Lane-6: 25UD; Lane-7: 30TD.

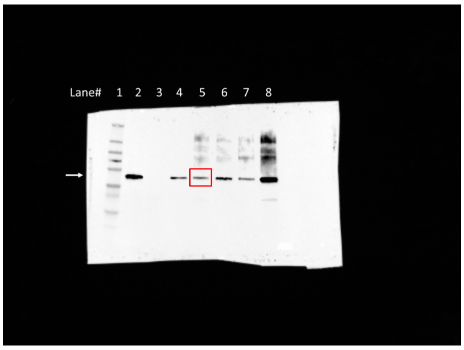

Supplementary Information

Supplementary Figure S6. The original, uncropped images of the blots correspond to Figure 1D (pancreas PI3K-p85α) in the manuscript. Please note that sample ID for example: 1LC indicates animal ID (#1) of the lean cat group (LC). Group abbreviations are: LC=lean cat; OW=overweight; UD=untreated diabetic; TD=treated diabetic. Blots/bands highlighted in red box represent the images shown in the manuscript.

Blot 3C: (from left to right) Lane-1: ladder; Lane-2: 6UD; Lane-3: 18TD; Lane-4: 8LC; Lane-5: 6OW; Lane-6: 8UD; Lane-7: 21TD; Lane-8: 9LC; Lane-9: 8OW; Lane-10: 11UD.

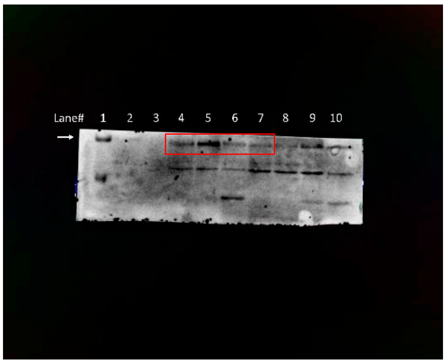

Supplementary Figure S7. The original, uncropped images of the blots correspond to Figure 1E (pancreas MAPK-p42/44) in the manuscript. Please note that sample ID for example: 1LC indicates animal ID (#1) of the lean cat group (LC). Group abbreviations are: LC=lean cat; OW=overweight; UD=untreated diabetic; TD=treated diabetic. Blots/bands highlighted in red box represent the images shown in the manuscript.

Blot 4C: (from left to right) Lane-1: ladder; Lane-2: 6UD; Lane-3: 18TD; Lane-4: 8LC; Lane-5: 6OW; Lane-6: 8UD; Lane-7: 21TD; Lane-8: 9LC; Lane-9: 8OW; Lane-10: 11UD.

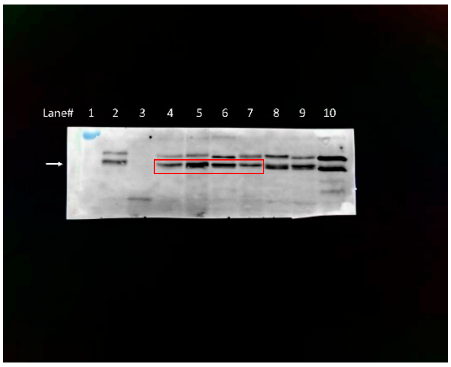

Supplementary Figure S8. The original, uncropped images of the blots correspond to Figure 1F (pancreas AKT) in the manuscript. Please note that sample ID for example: 1LC indicates animal ID (#1) of the lean cat group (LC). Group abbreviations are: LC=lean cat; OW=overweight; UD=untreated diabetic; TD=treated diabetic. Blots/bands highlighted in red box represent the images shown in the manuscript.

Blot 5C: (from left to right) Lane-1: ladder; Lane-2: 6UD; Lane-3: 18TD; Lane-4: 8LC; Lane-5: 6OW; Lane-6: 8UD; Lane-7: 21TD; Lane-8: 9LC; Lane-9: 8OW; Lane-10: 11UD.

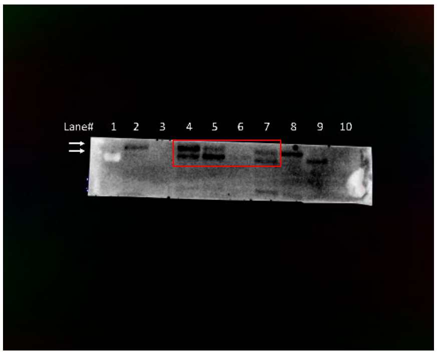

Supplementary Information

Supplementary Figure S9. The original, uncropped images of the blots correspond to normalization control (HSP70) for pancreas AKT and GLP-1R in the manuscript. Please note that sample ID for example: 1LC indicates animal ID (#1) of the lean cat group (LC). Group abbreviations are: LC=lean cat; OW=overweight; UD=untreated diabetic; TD=treated diabetic. Blots/bands highlighted in red box represent the images shown in the manuscript.

Blot 5I: (from left to right) Lane-1: ladder; Lane-2: 6UD; Lane-3: 18TD; Lane-4: 8LC; Lane-5: 6OW; Lane-6: 8UD; Lane-7: 21TD; Lane-8: 9LC; Lane-9: 8OW; Lane-10: 11UD.

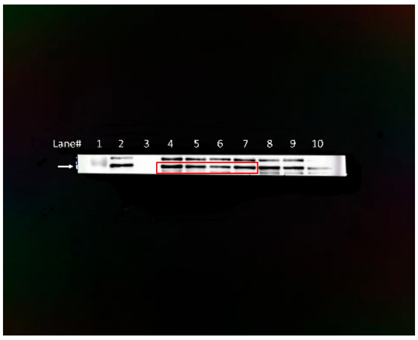

Supplementary Figure S10. The original, uncropped images of the blots correspond to Figure 1H (pancreas GLUT-1) in the manuscript. Please note that sample ID for example: 1LC indicates animal ID (#1) of the lean cat group (LC). Group abbreviations are: LC=lean cat; OW=overweight; UD=untreated diabetic; TD=treated diabetic. Blots/bands highlighted in red box represent the images shown in the manuscript.

Blot 6C: (from left to right) Lane-1: ladder; Lane-2: 6UD; Lane-3: 18TD; Lane-4: 8LC; Lane-5: 6OW; Lane-6: 8UD; Lane-7: 21TD; Lane-8: 9LC; Lane-9: 8OW; Lane-10: 11UD.

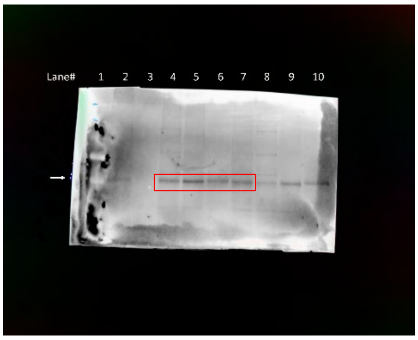

Supplementary Information

Supplementary Figure S11. The original, uncropped images of the blots correspond to Figure 1I (pancreas GLUT-2) in the manuscript. Please note that sample ID for example: 1LC indicates animal ID (#1) of the lean cat group (LC). Group abbreviations are: LC=lean cat; OW=overweight; UD=untreated diabetic; TD=treated diabetic. Blots 6A-F were stripped with Restore™ stripping buffer (cat# 21059, Thermo Scientific™) and reprobed with GLUT-2 and HSP70. Blots/bands highlighted in red box represent the images shown in the manuscript.

Blot 6I: (from left to right) Lane-1: ladder; Lane-2: 6UD; Lane-3: 18TD; Lane-4: 8LC; Lane-5: 6OW; Lane-6: 8UD; Lane-7: 21TD; Lane-8: 9LC; Lane-9: 8OW; Lane-10: 11UD.

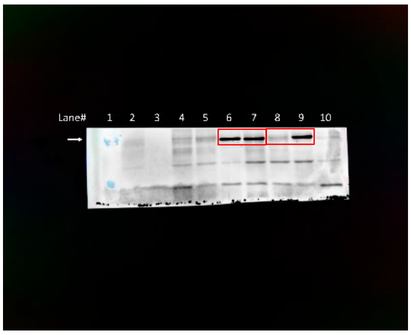

Supplementary Figure S12. Normalization control (HSP70) for pancreas GLUT-1 and GLUT-2. Please note that sample ID for example: 1LC indicates animal ID (#1) of the lean cat group (LC). Group abbreviations are: LC=lean cat; OW=overweight; UD=untreated diabetic; TD=treated diabetic. Blots/bands highlighted in red box represent the images shown in the manuscript.

Blot 6O: (from left to right) Lane-1: ladder; Lane-2: 6UD; Lane-3: 18TD; Lane-4: 8LC; Lane-5: 6OW; Lane-6: 8UD; Lane-7: 21TD; Lane-8: 9LC; Lane-9: 8OW; Lane-10: 11UD.

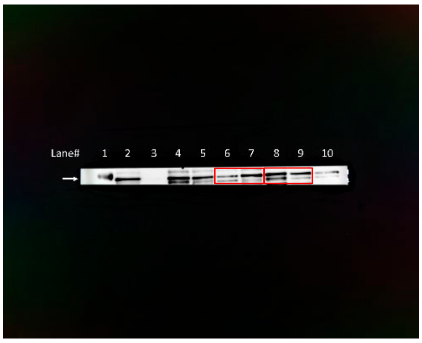

Supplementary Figure S13. The original, uncropped images of the blots correspond to Figure 1J (pancreas GLP-1R) in the manuscript. Please note that sample ID for example: 1LC indicates animal ID (#1) of the lean cat group (LC). Group abbreviations are: LC=lean cat; OW=overweight; UD=untreated diabetic; TD=treated diabetic. Blots/bands highlighted in red box represent the images shown in the manuscript.

Blot 7C: (from left to right) Lane-1: ladder; Lane-2: 6UD; Lane-3: 18TD; Lane-4: 8LC; Lane-5: 6OW; Lane-6: 8UD; Lane-7: 21TD; Lane-8: 9LC; Lane-9: 8OW; Lane-10: 11UD.

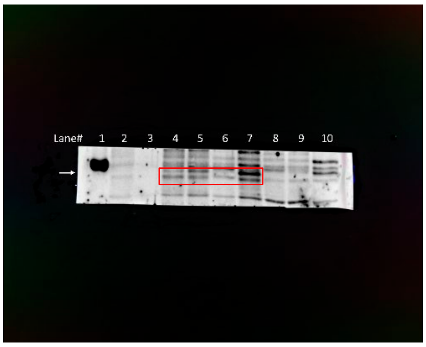

Supplementary Figure S14. The original, uncropped images of the blots correspond to Figure 1K (pancreas GIPR) in the manuscript. Please note that sample ID for example: 1LC indicates animal ID (#1) of the lean cat group (LC). Group abbreviations are: LC=lean cat; OW=overweight; UD=untreated diabetic; TD=treated diabetic. Blots/bands highlighted in red box represent the images shown in the manuscript.

Blot 8B: (from left to right) Lane-1: ladder; Lane-2: 3OW; Lane-3: 4UD; Lane-4: 10TD; Lane-5: 5LC; Lane-6: 4OW; Lane-7: 5UD; Lane-8: 14TD; Lane-9: 6LC; Lane-10: 5OW.

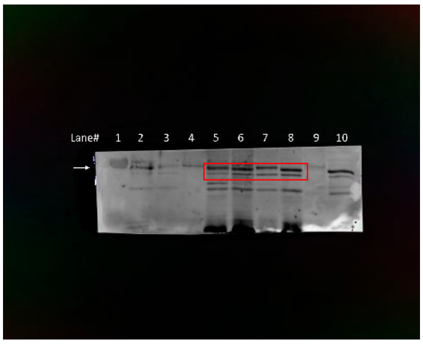

Normalization control (HSP70) for pancreas GIPR

Blot 8H: (from left to right) Lane-1: ladder; Lane-2: 3OW; Lane-3: 4UD; Lane-4: 10TD; Lane-5: 5LC; Lane-6: 4OW; Lane-7: 5UD; Lane-8: 14TD; Lane-9: 6LC; Lane-10: 5OW.

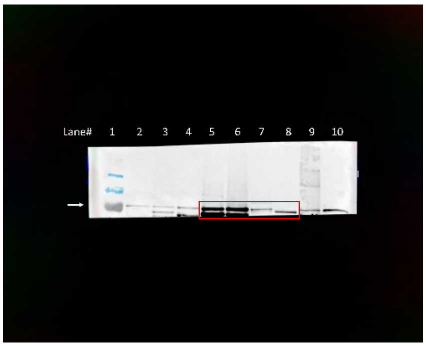

Supplementary Figure S15. The original, uncropped images of the blots correspond to Figure 2B (muscle insulin receptor) in the manuscript. Please note that sample ID for example: 3LC indicates animal ID (#3) of the lean cat group (LC). Group abbreviations are: LC=lean cat; OW=overweight; UD=untreated diabetic; TD=treated diabetic. Blots/bands highlighted in red box represent the images shown in the manuscript.

Blot 9A: (from left to right) Lane-1: ladder; Lane-2: 3LC; Lane-3: 3OW; Lane-4: 2UD; Lane-5: 1TD; Lane-6: 5LC; Lane-7: 4OW; Lane-8: 4UD; Lane-9: 5TD.

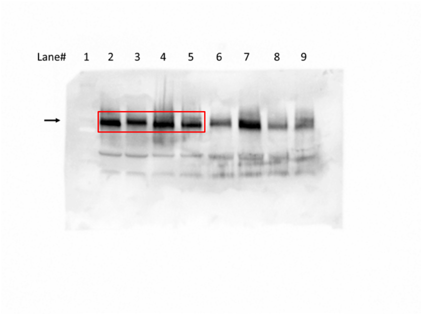

Normalization control (beta actin) for muscle insulin receptor

Blot 9D: (from left to right) Lane-1: ladder; Lane-2: 3LC; Lane-3: 3OW; Lane-4: 2UD; Lane-5: 1TD; Lane-6: 5LC; Lane-7: 4OW; Lane-8: 4UD; Lane-9: 5TD.

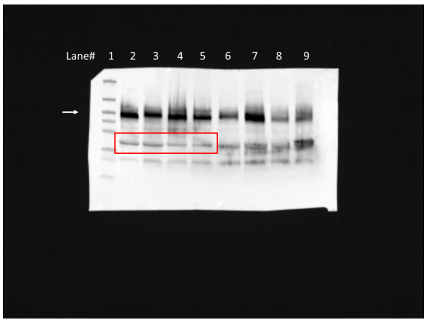

Supplementary Figure S16. The original, uncropped images of the blots correspond to Figure 2C (muscle PI3K-p85α) in the manuscript. Please note that sample ID for example: 3LC indicates animal ID (#3) of the lean cat group (LC). Group abbreviations are: LC=lean cat; OW=overweight; UD=untreated diabetic; TD=treated diabetic. Blots/bands highlighted in red box represent the images shown in the manuscript.

Blot 10A: (from left to right) Lane-1: ladder; Lane-2: 3LC; Lane-3: 3OW; Lane-4: 2UD; Lane-5: 1TD; Lane-6: 5LC; Lane-7: 4OW; Lane-8: 4UD; Lane-9: 5TD.

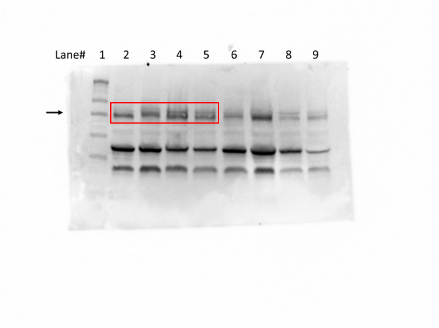

Normalization control (beta actin) for muscle PI3K-p85α

Blot 10D: (from left to right) Lane-1: ladder; Lane-2: 1LC3LC; Lane-3: 3OW; Lane-4: 2UD; Lane-5: 1TD; Lane-6: 5LC; Lane-7: 4OW; Lane-8: 4UD; Lane-9: 5TD.

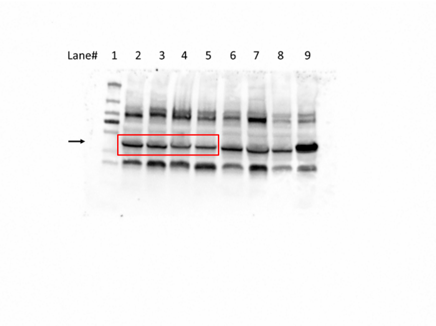

Supplementary Figure S17. The original, uncropped images of the blots correspond to Figure 2D (muscle GLUT-4) in the manuscript. Please note that sample ID for example: 1LC indicates animal ID (#1) of the lean cat group (LC). Group abbreviations are: LC=lean cat; OW=overweight; UD=untreated diabetic; TD=treated diabetic. Blots/bands highlighted in red box represent the images shown in the manuscript.

Blot 23B: (from left to right) Lane-1: ladder; Lane-2: 5UD; Lane-3: 14TD; Lane-4: 8LC; Lane-5: 8OW; Lane-6: 6UD; Lane-7: 18TD; Lane-8: 9LC; Lane-9: 9OW; Lane-10: 8UD; Lane-11: 21TD; Lane-12: 10LC; Lane-13: 10OW; Lane-14: 11UD; Lane-15: 27TD.

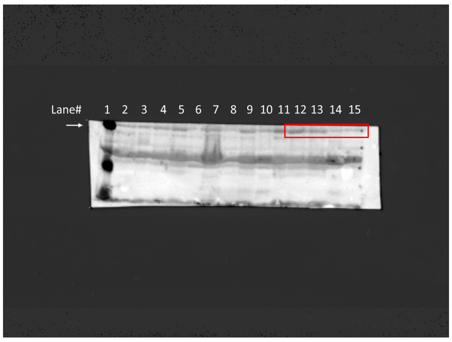

Normalization control (HSP70) for muscle GLUT-4

Blot 23F: (from left to right) Lane-1: ladder; Lane-2: 5UD; Lane-3: 14TD; Lane-4: 8LC; Lane-5: 8OW; Lane-6: 6UD; Lane-7: 18TD; Lane-8: 9LC; Lane-9: 9OW; Lane-10: 8UD; Lane-11: 21TD; Lane-12: 10LC; Lane-13: 10OW; Lane-14: 11UD; Lane-15: 27TD.

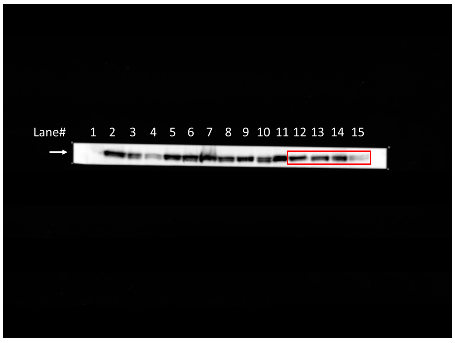

Supplementary Figure S18. The original, uncropped images of the blots correspond to Figure 2E (muscle GLP-1 receptor) in the manuscript. Please note that sample ID for example: 1LC indicates animal ID (#1) of the lean cat group (LC). Group abbreviations are: LC=lean cat; OW=overweight; UD=untreated diabetic; TD=treated diabetic. Blots/bands highlighted in red box represent the images shown in the manuscript.

Blot 24B: (from left to right) Lane-1: ladder; Lane-2: 5UD; Lane-3: 14TD; Lane-4: 8LC; Lane-5: 8OW; Lane-6: 6UD; Lane-7: 18TD; Lane-8: 9LC; Lane-9: 9OW; Lane-10: 8UD; Lane-11: 21TD; Lane-12: 10LC; Lane-13: 10OW; Lane-14: 11UD; Lane-15: 27TD.

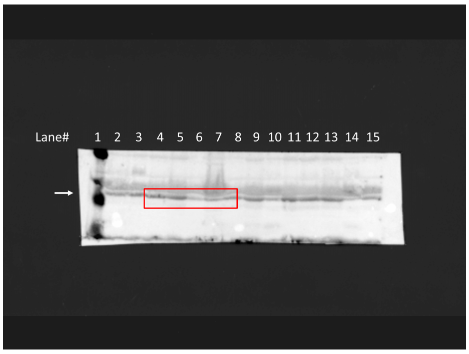

Normalization control (HSP70) for muscle GLP-1 receptor

Blot 23F: (from left to right) Lane-1: ladder; Lane-2: 5UD; Lane-3: 14TD; Lane-4: 8LC; Lane-5: 8OW; Lane-6: 6UD; Lane-7: 18TD; Lane-8: 9LC; Lane-9: 9OW; Lane-10: 8UD; Lane-11: 21TD; Lane-12: 10LC; Lane-13: 10OW; Lane-14: 11UD; Lane-15: 27TD.

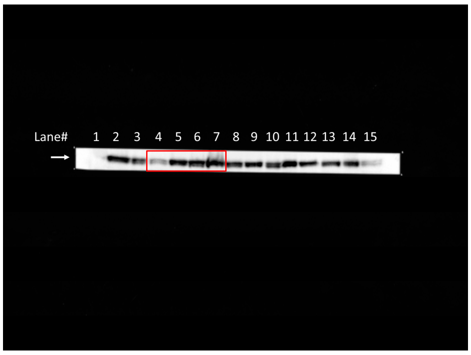

Supplementary Figure S19. The original, uncropped images of the blots correspond to Figure 3B (liver insulin receptor) in the manuscript. Please note that sample ID for example: 1LC indicates animal ID (#1) of the lean cat group (LC). Group abbreviations are: LC=lean cat; OW=overweight; UD=untreated diabetic; TD=treated diabetic. Blots/bands highlighted in red box represent the images shown in the manuscript.

Blot 13A.1: The original, uncropped blot of liver insulin receptor consisting lean (LC), overweight (OW), untreated diabetic (UD) and treated diabetic (TD) samples. (From left to right) Lane-1: ladder; Lane-2: 4LC; Lane-3: 9OW; Lane-4: 2UD; Lane-5: 1TD; Lane-6: 5LC; Lane-7: 9OW; Lane-8: 2UD; Lane-9: 1TD.

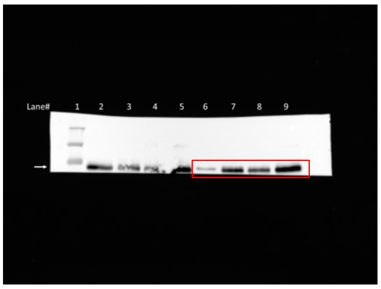

Normalization control (beta actin) for liver insulin receptor

Blot 13G.1: The original, uncropped blot of internal control (beta actin) for liver insulin receptor consisting lean (LC), overweight (OW), untreated diabetic (UD) and treated diabetic (TD) samples. (From left to right) Lane-1: ladder; Lane-2: 4LC; Lane-3: 9OW; Lane-4: 2UD; Lane-5: 1TD; Lane-6: 5LC; Lane-7: 9OW; Lane-8: 2UD; Lane-9: 1TD.

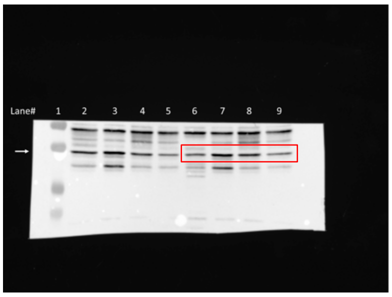

Supplementary Information

Supplementary Figure S20. The original, uncropped images of the blots correspond to Figure 3C (liver IRS-1) in the manuscript. Please note that sample ID for example: 1LC indicates animal ID (#1) of the lean cat group (LC). Group abbreviations are: LC=lean cat; OW=overweight; UD=untreated diabetic; TD=treated diabetic. Blots/bands highlighted in red box represent the images shown in the manuscript.

Blot 14A.1: The original, uncropped blot of liver IRS-1 consisting of lean (LC), overweight (OW), untreated diabetic (UD) and treated diabetic (TD) samples. (From left to right) Lane-1: ladder; Lane-2: 3LC; Lane-3: 3OW; Lane-4: 2UD; Lane-5: 1TD; Lane-6: 1LC; Lane-7: 10OW; Lane-8: 12UD; Lane-9: 18TD.

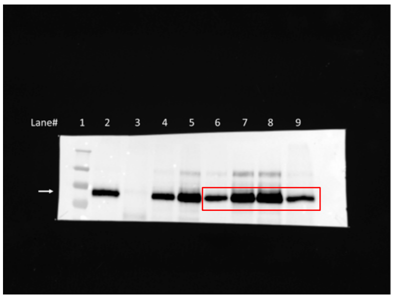

Normalization control (beta actin) for liver IRS-1

Blot 14G.1: The original, uncropped blot of internal control (beta actin) for liver IRS-1 consisting lean (LC), overweight (OW), untreated diabetic (UD) and treated diabetic (TD) samples. (From left to right) Lane-1: ladder; Lane-2: 3LC; Lane-3: 3OW; Lane-4: 2UD; Lane-5: 1TD; Lane-6: 1LC; Lane-7: 10OW; Lane-8: 12UD; Lane-9: 18TD.

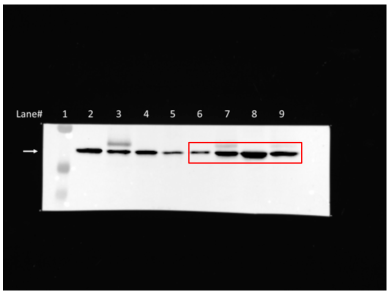

Supplementary Figure S21. The original, uncropped images of the blots correspond to Figure 3D (liver PI3K-p85α) in the manuscript. Please note that sample ID for example: 1LC indicates animal ID (#1) of the lean cat group (LC). Group abbreviations are: LC=lean cat; OW=overweight; UD=untreated diabetic; TD=treated diabetic. Blots/bands highlighted in red box represent the images shown in the manuscript.

Blot 15A.1: The original, uncropped blot of liver PI3K-p85α consisting of lean (LC), overweight (OW), untreated diabetic (UD) and treated diabetic (TD) samples. (From left to right) Lane-1: ladder; Lane-2: 3LC; Lane-3: 10OW; Lane-4: 2UD; Lane-5: 1TD; Lane-6: 15LC; Lane-7: 17OW; Lane-8: 12UD; Lane-9: 1TD.

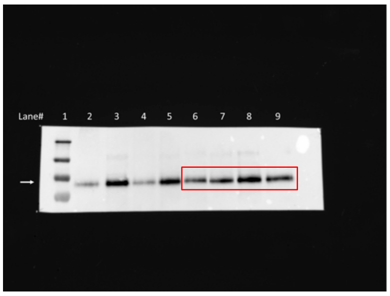

Normalization control (beta actin) for liver PI3K-p85α

Blot 15F.1: The original, uncropped blot of internal control (beta actin) for liver PI3K-p85α consisting lean (LC), overweight (OW), untreated diabetic (UD) and treated diabetic (TD) samples. (From left to right) Lane-1: ladder; Lane-2: 3LC; Lane-3: 10OW; Lane-4: 2UD; Lane-5: 1TD; Lane-6: 15LC; Lane-7: 17OW; Lane-8: 12UD; Lane-9: 1TD.

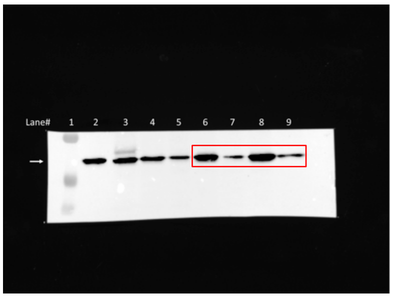

Supplementary Figure S22. The original, uncropped images of the blots correspond to Figure 3E (liver MAPK-p42/44) in the manuscript. Please note that sample ID for example: 1LC indicates animal ID (#1) of the lean cat group (LC). Group abbreviations are: LC=lean cat; OW=overweight; UD=untreated diabetic; TD=treated diabetic. Blots/bands highlighted in red box represent the images shown in the manuscript.

Blot 16A: (from left to right) Lane-1: ladder; Lane-2: 1LC; Lane-3: 1OW; Lane-4: 2UD; Lane-5: 1TD; Lane-6: 3LC; Lane-7: 2OW; Lane-8: 3UD; Lane-9: 9TD; Lane-10: 4LC.

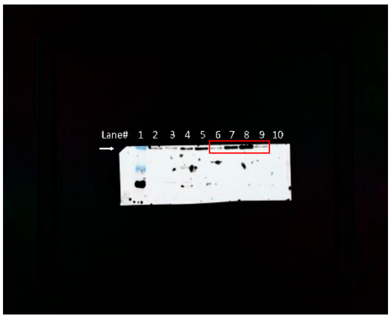

Normalization control (beta actin) for liver MAPK-p42/44

Blot 16G: (from left to right) Lane-1: ladder; Lane-2: 1LC; Lane-3: 1OW; Lane-4: 2UD; Lane-5: 1TD; Lane-6: 3LC; Lane-7: 2OW; Lane-8: 3UD; Lane-9: 9TD; Lane-10: 4LC.

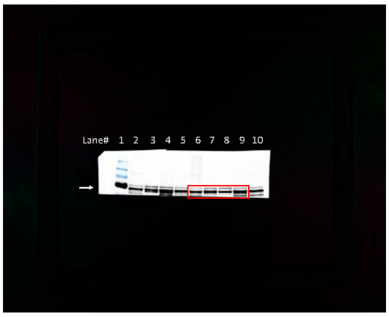

Supplementary Figure S23. The original, uncropped images of the blots correspond to Figure 3F (liver AKT) in the manuscript. Please note that sample ID for example: 1LC indicates animal ID (#1) of the lean cat group (LC). Group abbreviations are: LC=lean cat; OW=overweight; UD=untreated diabetic; TD=treated diabetic. Blots/bands highlighted in red box represent the images shown in the manuscript.

Blot 17C: (from left to right) Lane-1: ladder; Lane-2: 6UD; Lane-3: 18TD; Lane-4: 8LC; Lane-5: 6OW; Lane-6: 8UD; Lane-7: 21TD; Lane-8: 9LC; Lane-9: 8OW; Lane-10: 11UD.

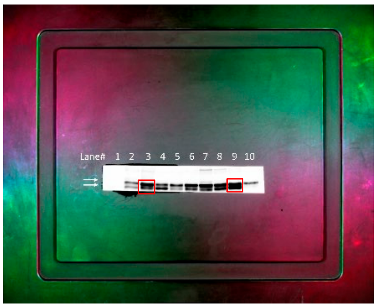

Blot 17D: (from left to right) Lane-1: ladder; Lane-2: 27TD; Lane-3: 10LC; Lane-4: 9OW; Lane-5: 12UD; Lane-6: 11LC; Lane-7: 10OW; Lane-8: 16UD; Lane-9: 13LC; Lane-10: 11OW.

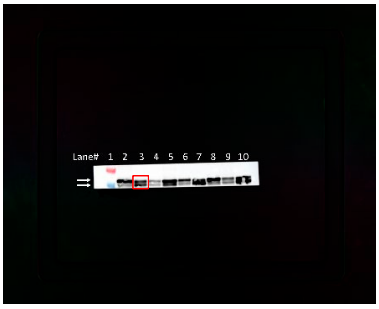

Blot 17E: (from left to right) Lane-1: ladder; Lane-2: 17UD; Lane-3: 14LC; Lane-4: 13OW; Lane-5: 19UD; Lane-6: 15LC; Lane-7: 14OW; Lane-8: 15AUD; Lane-9: 18LC; Lane-10: 17OW.

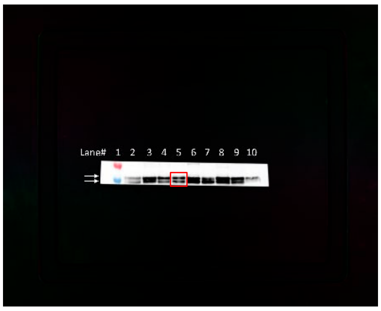

Supplementary Information

Supplementary Figure S24. Normalization control (beta actin) for liver AKT and ACC in Figure 3. Please note that sample ID for example: 1LC indicates animal ID (#1) of the lean cat group (LC). Group abbreviations are: LC=lean cat; OW=overweight; UD=untreated diabetic; TD=treated diabetic. Blots/bands highlighted in red box represent the images shown in the manuscript.

Blot 17I: (from left to right) Lane-1: ladder; Lane-2: 6UD; Lane-3: 18TD; Lane-4: 8LC; Lane-5: 6OW; Lane-6: 8UD; Lane-7: 21TD; Lane-8: 9LC; Lane-9: 8OW; Lane-10: 11UD.

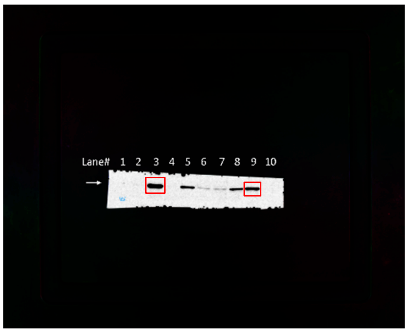

Blot 17J: (from left to right) Lane-1: ladder; Lane-2: 27TD; Lane-3: 10LC; Lane-4: 9OW; Lane-5: 12UD; Lane-6: 11LC; Lane-7: 10OW; Lane-8: 16UD; Lane-9: 13LC; Lane-10: 11OW.

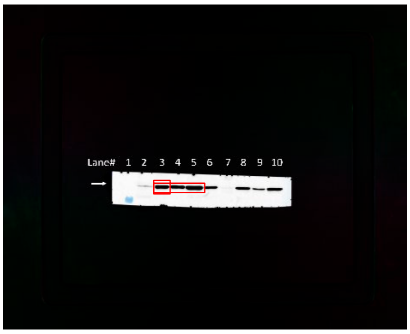

Blot 17K: (from left to right) Lane-1: ladder; Lane-2: 17UD; Lane-3: 14LC; Lane-4: 13OW; Lane-5: 19UD; Lane-6: 15LC; Lane-7: 14OW; Lane-8: 15AUD; Lane-9: 18LC; Lane-10: 17OW.

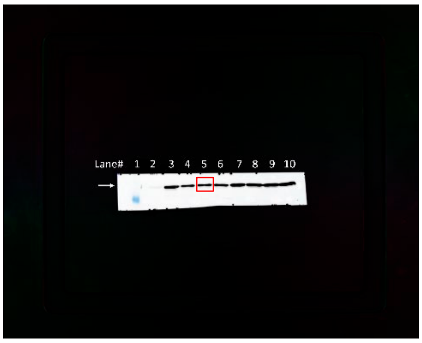

Supplementary Figure S25. The original, uncropped images of the blots correspond to Figure 3G (liver GLP-1R) in the manuscript. Please note that sample ID for example: 1LC indicates animal ID (#1) of the lean cat group (LC). Group abbreviations are: LC=lean cat; OW=overweight; UD=untreated diabetic; TD=treated diabetic. Blots/bands highlighted in red box represent the images shown in the manuscript.

Blot 18B: (from left to right) Lane-1: ladder; Lane-2: 3OW; Lane-3: 4UD; Lane-4: 10TD; Lane-5: 5LC; Lane-6: 4OW; Lane-7: 5UD; Lane-8: 14TD; Lane-9: 6LC; Lane-10: 5OW.

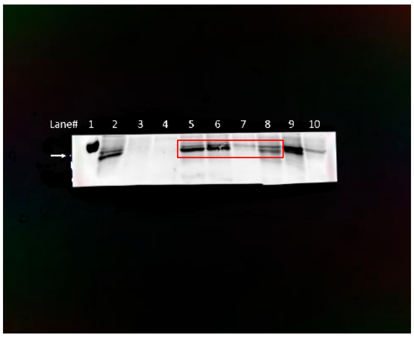

Normalization control (beta actin) for liver GLP-1R

Blot 18H: (from left to right) Lane-1: ladder; Lane-2: 3OW; Lane-3: 4UD; Lane-4: 10TD; Lane-5: 5LC; Lane-6: 4OW; Lane-7: 5UD; Lane-8: 14TD; Lane-9: 6LC; Lane-10: 5OW.

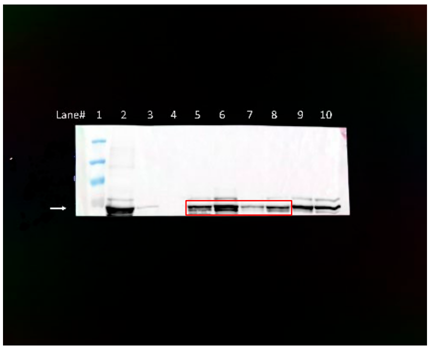

Supplementary Figure S26. The original, uncropped images of the blots correspond to Figure 3H (liver ACC) in the manuscript. Please note that sample ID for example: 1LC indicates animal ID (#1) of the lean cat group (LC). Group abbreviations are: LC=lean cat; OW=overweight; UD=untreated diabetic; TD=treated diabetic. Blots/bands highlighted in red box represent the images shown in the manuscript.

Blot 19C: (from left to right) Lane-1: ladder; Lane-2: 6UD; Lane-3: 18TD; Lane-4: 8LC; Lane-5: 6OW; Lane-6: 8UD; Lane-7: 21TD; Lane-8: 9LC; Lane-9: 8OW; Lane-10: 11UD.

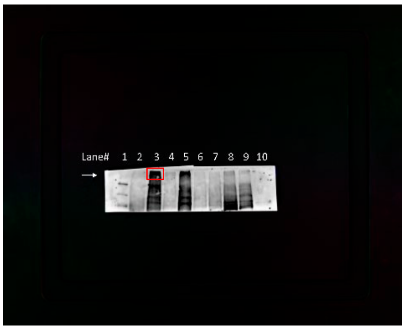

Blot 19D: (from left to right) Lane-1: ladder; Lane-2: 27TD; Lane-3: 10LC; Lane-4: 9OW; Lane-5: 12UD; Lane-6: 11LC; Lane-7: 10OW; Lane-8: 16UD; Lane-9: 13LC; Lane-10: 11OW.

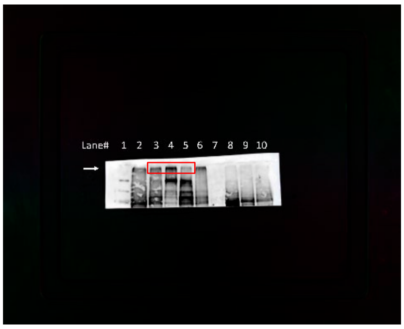

Supplementary Information

Supplementary Figure S27. The original, uncropped images of the blots correspond to Figure 3I (liver FAS) in the manuscript. Please note that sample ID for example: 1LC indicates animal ID (#1) of the lean cat group (LC). Group abbreviations are: LC=lean cat; OW=overweight; UD=untreated diabetic; TD=treated diabetic. Blots/bands highlighted in red box represent the images shown in the manuscript.

Blot 20A: (from left to right) Lane-1: ladder; Lane-2: 1LC; Lane-3: 1OW; Lane-4: 2UD; Lane-5: 1TD; Lane-6: 3LC; Lane-7: 2OW; Lane-8: 3UD; Lane-9: 9TD; Lane-10: 4LC.

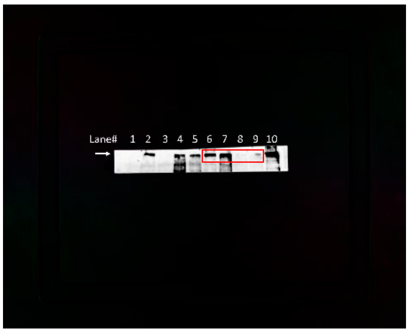

Supplementary Information

Supplementary Figure S28. The original, uncropped images of the blots correspond to Figure 3J (liver HSL) in the manuscript. Please note that sample ID for example: 1LC indicates animal ID (#1) of the lean cat group (LC). Group abbreviations are: LC=lean cat; OW=overweight; UD=untreated diabetic; TD=treated diabetic. Blots/bands highlighted in red box represent the images shown in the manuscript.

Blot 21A: (from left to right) Lane-1: ladder; Lane-2: 1LC; Lane-3: 1OW; Lane-4: 2UD; Lane-5: 1TD; Lane-6: 3LC; Lane-7: 2OW; Lane-8: 3UD; Lane-9: 9TD; Lane-10: 4LC.

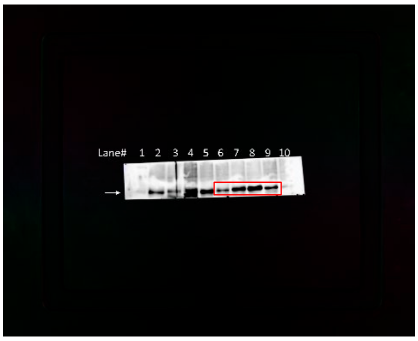

Supplementary Figure S29. Normalization control (HSP70) for liver FAS and HSL in Figure 3. Please note that sample ID for example: 1LC indicates animal ID (#1) of the lean cat group (LC). Group abbreviations are: LC=lean cat; OW=overweight; UD=untreated diabetic; TD=treated diabetic. Blots/bands highlighted in red box represent the images shown in the manuscript.

Blot 21G: (from left to right) Lane-1: ladder; Lane-2: 1LC; Lane-3: 1OW; Lane-4: 2UD; Lane-5: 1TD; Lane-6: 3LC; Lane-7: 2OW; Lane-8: 3UD; Lane-9: 9TD; Lane-10: 4LC.

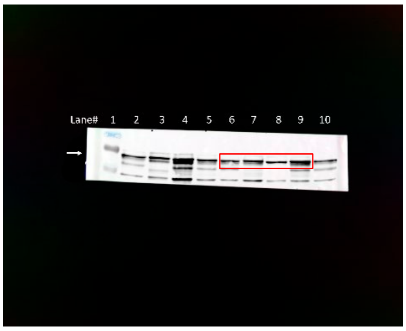

Supplementary Figure S30. The original, uncropped images of the blots correspond to Figure 1G (pancreas phospho-AKT) in the manuscript. Please note that sample ID for example: 1LC indicates animal ID (#1) of the lean cat group (LC). Group abbreviations are: LC=lean cat; OW=overweight; UD=untreated diabetic; TD=treated diabetic. Blots/bands highlighted in red box represent the images shown in the manuscript.

Blot 22A: (from left to right) Lane-1: ladder; Lane-2: 1LC; Lane-3: 1OW; Lane-4: 2UD; Lane-5: 1TD; Lane-6: 3LC; Lane-7: 2OW; Lane-8: 3UD; Lane-9: 9TD; Lane-10: 4LC; Lane-11: 3OW; Lane-12: 4UD; Lane-13: 10TD; Lane-14: 5LC; Lane-15: 4OW.

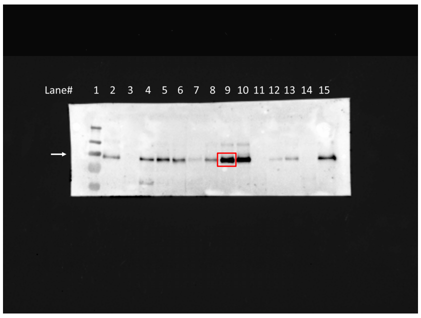

Blot 22C: (from left to right) Lane-1: ladder; Lane-2: 10LC; Lane-3: 9OW; Lane-4: 12UD; Lane-5: 11LC; Lane-6: 10OW; Lane-7: 16UD; Lane-8: 13LC; Lane-9: 11OW; Lane-10: 17UD; Lane-11: 14LC; Lane-12: 13OW; Lane-13: 19UD; Lane-14: 15LC; Lane-15: 14OW.

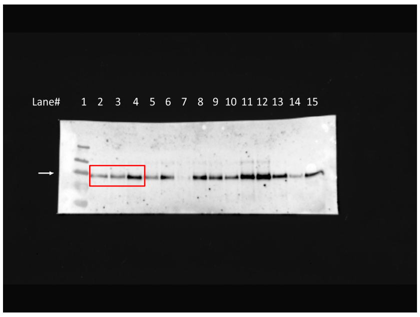

Supplementary Figure S31. The original, uncropped images of the blots correspond to Figure 2F (muscle MAPK-p42/44) in the manuscript. Please note that sample ID for example: 1LC indicates animal ID (#1) of the lean cat group (LC). Group abbreviations are: LC=lean cat; OW=overweight; UD=untreated diabetic; TD=treated diabetic. Blots/bands highlighted in red box represent the images shown in the manuscript.

Blot 25A: (from left to right) Lane-1: ladder; Lane-2: 3LC; Lane-3: 1OW; Lane-4: 2UD; Lane-5: 1TD; Lane-6: 4LC; Lane-7: 2OW; Lane-8: 3UD; Lane-9: 9TD; Lane-10: 5LC; Lane-11: 3OW; Lane-12: 4UD; Lane-13: 10TD; Lane-14: 6LC; Lane-15: 4OW.

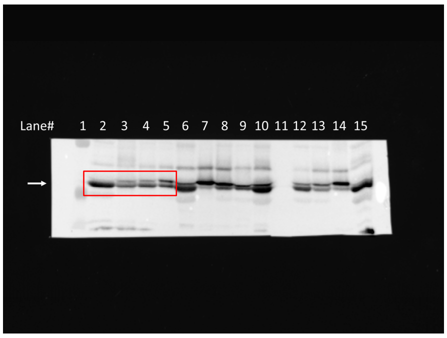

Normalization control (HSP70) for muscle MAPK-p42/44

Blot 25E: (from left to right) Lane-1: ladder; Lane-2: 3LC; Lane-3: 1OW; Lane-4: 2UD; Lane-5: 1TD; Lane-6: 4LC; Lane-7: 2OW; Lane-8: 3UD; Lane-9: 9TD; Lane-10: 5LC; Lane-11: 3OW; Lane-12: 4UD; Lane-13: 10TD; Lane-14: 6LC; Lane-15: 4OW.

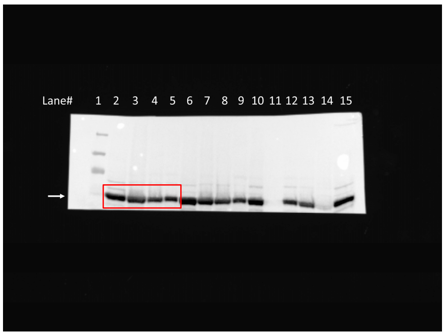

Supplementary Figure S32. The original, uncropped images of the blots correspond to Figure 3I (liver phospho-ACC) in the manuscript. Please note that sample ID for example: 1LC indicates animal ID (#1) of the lean cat group (LC). Group abbreviations are: LC=lean cat; OW=overweight; UD=untreated diabetic; TD=treated diabetic. Blots/bands highlighted in red box represent the images shown in the manuscript.

Blot 26A: (from left to right) Lane-1: ladder; Lane-2: 1LC; Lane-3: 1OW; Lane-4: 2UD; Lane-5: 1TD; Lane-6: 2LC; Lane-7: 2OW; Lane-8: 3UD; Lane-9: 9TD; Lane-10: 3LC; Lane-11: 3OW; Lane-12: 4UD; Lane-13: 10TD; Lane-14: 4LC; Lane-15: 4OW.

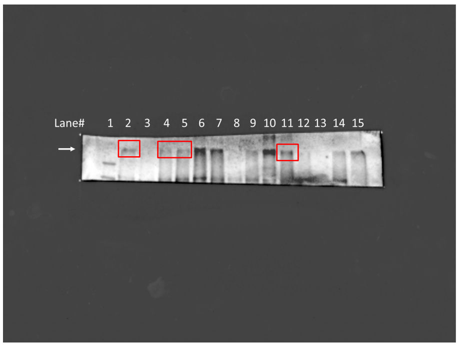

Normalization control (beta actin) for liver phospho-ACC

Blot 26E: (from left to right) Lane-1: ladder; Lane-2: 1LC; Lane-3: 1OW; Lane-4: 2UD; Lane-5: 1TD; Lane-6: 2LC; Lane-7: 2OW; Lane-8: 3UD; Lane-9: 9TD; Lane-10: 3LC; Lane-11: 3OW; Lane-12: 4UD; Lane-13: 10TD; Lane-14: 4LC; Lane-15: 4OW.

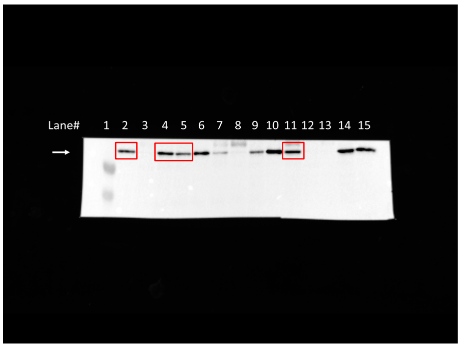

Supplementary Figure S33. The original, uncropped images of the blots correspond to Figure 3L (liver phospho-HSL) in the manuscript. Please note that sample ID for example: 1LC indicates animal ID (#1) of the lean cat group (LC). Group abbreviations are: LC=lean cat; OW=overweight; UD=untreated diabetic; TD=treated diabetic. Blots/bands highlighted in red box represent the images shown in the manuscript.

Blot 27A: (from left to right) Lane-1: ladder; Lane-2: 1LC; Lane-3: 1OW; Lane-4: 2UD; Lane-5: 1TD; Lane-6: 2LC; Lane-7: 2OW; Lane-8: 3UD; Lane-9: 9TD; Lane-10: 3LC; Lane-11: 3OW; Lane-12: 4UD; Lane-13: 10TD; Lane-14: 4LC; Lane-15: 4OW.

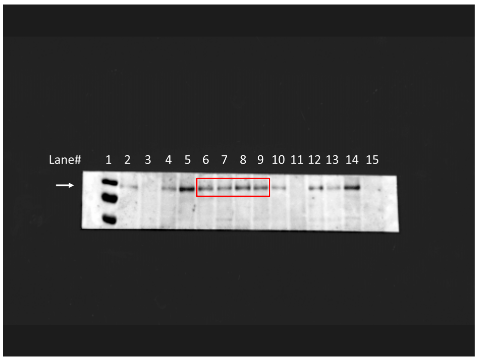

Supplement: Supplementary file 1 [file ijms-25-13195-s001.zip › ijms-3318551-supplementary.pdf]
